# Osmotic tolerance of avian erythrocytes to complete hemolysis in solute free water

**DOI:** 10.1038/s41598-019-44487-7

**Published:** 2019-05-28

**Authors:** Snigdha Singh, Nisha Ponnappan, Anand Verma, Aditya Mittal

**Affiliations:** 1 Kusuma School of Biological Sciences, Indian Institute of Technology Delhi (IIT Delhi), Hauz Khas, New Delhi 110016 India; 2 National Institute of Pathology – Indian Council of Medical Research (ICMR), New Delhi, 110029 India; 3 Present Address: Green trace consulting Pvt Ltd, Delhi, 110096 India

**Keywords:** Cellular microbiology, Porins, Ion transport, Physiology, Diagnostic markers

## Abstract

Osmotic behavior of erythrocytes is not only important clinically, but is also significant in understanding of material transport across biological membranes. It is most commonly studied through fragiligrams – plots of the degree of hemolysis as a function of extracellular osmolarity. A fundamental assumption in experimental and theoretical studies on osmolarity driven transport of water across the plasma membranes of all cells is the sigmoidal nature of their osmotic behavior. Sigmoidal data is mathematically monotonic showing either a decreasing only or an increasing only trend, but not both, within certain thresholds; beyond these thresholds the data is asymptotic or flat. Fragiligrams of erythrocytes are usually sigmoidal, with maximal hemolysis in plain solute-free water and often up to a certain extracellular hypotonic environment. In this work, we report a new discovery of non-monotonic osmotic behavior of avian erythrocytes. In contrast to the expected monotonic fragiligrams obtained for mammalian erythrocytes, fragiligrams of avian erythrocytes show non-monotonic curves. Maximal hemolysis of avian erythrocytes was not observed at the most hypotonic conditions – instead, maximal hemolysis was observed at mild hypotonic conditions. Hemolysis of avian erythrocytes first increases then decreases with increasing extracellular osmolarity. We also report that the non-monotonic fragiligrams of chicken erythrocytes are converted to the expected monotonic sigmoids subsequent to controlled extracellular trypsinization. While possibly having profound evolutionary implications for vertebrates, the findings reported in this work have a direct impact on understanding of avian physiology. Our results also compel revisiting of experimental and theoretical models for understanding material transport across biological membranes under different osmotic conditions.

## Introduction

Since the invention of the microscope about 350 years ago, red blood cells (RBCs, also called erythrocytes) have played a central role in experimental and clinical biology. RBCs are arguably the most utilized natural model system for understanding various aspects of the unit of life i.e. a cell. Use of RBCs continues to specifically contribute towards gaining biophysical insights into assembly and functioning of biological membranes. For example, studies utilizing RBCs continue to result in fundamental understanding of elastic behavior and stability of biological membranes through experimental observations on osmotic hemolysis^1–4^. The ease of encapsulating soluble fluorophores in RBCs by mild hypotonic shock allows them to also serve as important model systems in understanding the dynamics of cellular membrane remodeling, e.g. during protein-mediated membrane fusion^5–8^. The availability of optical signatures in form of intracellular hemoglobin has also allowed the use of RBCs in exploring biosynthesis pathways for, and purification of, proteins from cells^9–12^. Release of hemoglobin with or without complete cell lyses along with exchange of various chemical species across RBC plasma membranes in different conditions have formed the basis of understanding material transport in living cells^13–16^. Being a key common cellular feature in all vertebrates, RBCs also feature in studies aimed at gaining of evolutionary^17–19^ and developmental^19,20^ insights into, and understanding comparative physiology^21–23^ of, different vertebrate species. Studies utilizing RBCs often rely upon observations of their morphological characteristics in different physico-chemical conditions. Mammalian RBCs are known to have a “standard” discoid-doughnut-shape in natural conditions. Avian erythrocytes appear slightly different due to the presence of intracellular organelles, e.g. mitochondria^19^ and nucleus^24^. Beyond morphology, the sensitivity of RBCs to different extracellular osmotic environments has emerged as a key experimentally measured feature over the last century^1,25–27^. This sensitivity is parameterized as osmotic fragility. Osmotic fragility measurements of RBCs under different physiological conditions, including different kinds of stresses, in vertebrates continue to evolve towards having more clinical and diagnostic significance in different species^28–31^.

Osmotic fragility data of RBCs is generally presented in terms of “fragiligrams”. The degree or percentage of hemolysis, measured in terms of hemoglobin released due to hemolysis, is plotted as a function of extracellular osmolyte concentration. The basis of interpretation of fragiligrams is in the van’t Hoff equation relating osmotic pressure experienced by the RBC plasma membrane due to the difference in intra- and extra- cellular environments (osmolarities). The hemolytic release of hemoglobin from RBCs as a function of increasing extracellular osmolyte concentration is expected to follow a sigmoidal curve. The most hypotonic environment, i.e. water without any solutes, is expected to result in the largest amount of hemolysis, with the magnitude of hemolysis decreasing with increasing extracellular osmolarity. Mathematically the sigmoidal fragiligrams of RBCs are monotonic functions^32–35^. Monotonic data/curves show either a decreasing only or an increasing only trend, but not both, i.e. there is no change in the sign of first derivative of the data/curves. Hemolytic data in fragiligrams is expected to be and modeled as monotonic sigmoidal as a function of increasing extracellular osmolyte concentration. Maximal hemolysis is expected to be, and observed, in absence of any solute/osmolyte. Often, this maximal hemolysis is also observed up to very dilute extracellular osmolyte concentrations, i.e. up to a “hypotonic” threshold. This is followed by a linear/exponential-appearing decrease in the degree of hemolysis beyond the hypotonic threshold. Finally, no further hemolysis is observed at high osmolyte concentrations – the cells do not lyse, rather they shrink due to hypertonicity.

In this work, we report a new discovery of non-monotonic osmotic behavior of avian erythrocytes. Fragiligrams of chicken RBCs show non-monotonic curves, in contrast to the usual monotonic fragiligrams obtained for mammalian RBCs. For chicken RBCs, maximal hemolysis was not observed at the most hypotonic conditions (e.g. extracellular solutions were only water and/or were having lowest osmolyte concentrations) – instead, maximal hemolysis was observed at mild hypotonic conditions. The resulting fragiligram data shows an increase in hemolysis from most hypotonic to mild hypotonic extracellular conditions followed by a decrease in hemolysis at higher osmolyte concentrations. The non-monotonic fragiligrams were also supported by microscopic observations that showed maximal hemolysis only at mild hypotonic conditions and not at most hypotonic conditions.

To probe into the non-monotonic osmotic behavior of chicken RBCs, we explored literature relevant to different aspects of transport across cellular plasma membranes. Broadly, the overall integrity of cellular structures is maintained via cytoskeleton, plasma membrane lipids, transmembrane proteins. While cellular deformability has been explicitly shown to depend on cytoskeletal proteins^36^, lipid compositions and lengths of trans-membrane domains of the membrane proteins have shown to affect physical properties such as curvature^37,38^ and thickness^39,40^ of biological membranes. The transport of ions and water (and osmotic behavior by inference) has been well documented to be regulated specifically through ion- and water- channels^15,41,42^. Interestingly, we found that presence of inactive/non-functional aquaporins (membrane proteins that allow transport of water and sometimes other solutes) has been reported specifically in chicken RBCs^41^. These aquaporins are active in mammalian RBCs.

The above led to the obvious possibility of the inactive/non-functional aquaporins in chicken RBCs being responsible for their non-monotonic osmotic behavior. If this was the case, then activation of these aquaporins could result in osmotic behavior of chicken RBCs following the expected monotonic sigmoidal nature. In search of literature on possible ways to “activate” channels in plasma membranes, we found that extracellular protease treatment has been shown to affect transport of material (specific solutes including ions) across plasma membranes in some cell systems^43^. The observed effects are presumably due to partial or complete cleaving of ectodomains of plasma membrane proteins (specifically channel proteins, but possibly others also) by proteases. Thus, we hypothesized that cleaving ectodomains of plasma membrane proteins, including all aquaporins and other channels, of chicken RBCs may result in conversion of their non-monotonic osmotic behavior to that normally expected. To test the hypothesis, we used extracellular treatment of trypsin to shave trypsin-sensitive ectodomains of plasma membrane proteins. We report that controlled trypsinization of the chicken RBC surface leads to the usually expected monotonic sigmoidal fragiligrams.

Finally, it is important to point out that our finding of non-monotonic osmotic behavior of chicken RBCs is applicable to avian RBCs in general (see the section Non-monotonic Osmotic Fragility of avian RBCs in saline in “Results”). The discovery of non-monotonic fragiligrams of avian RBCs is expected to have a direct impact on understanding of avian physiology, especially in relation to physiology of other vertebrates. Additionally, this finding possibly has profound implications in our understanding of evolution of vertebrates (avian, amphibian, reptilian and mammalian) especially in terms of survival in different salt-water conditions. Protease-dependent conversion of the non-monotonic osmotic behavior of avian RBCs (to monotonic) also has evolutionary and developmental implications towards our understanding of not only acclimatization but also adaptation by birds to different aqueous environments.

## Results

### Expected osmotic fragility of mammalian RBCs in saline

**Figure 1:**
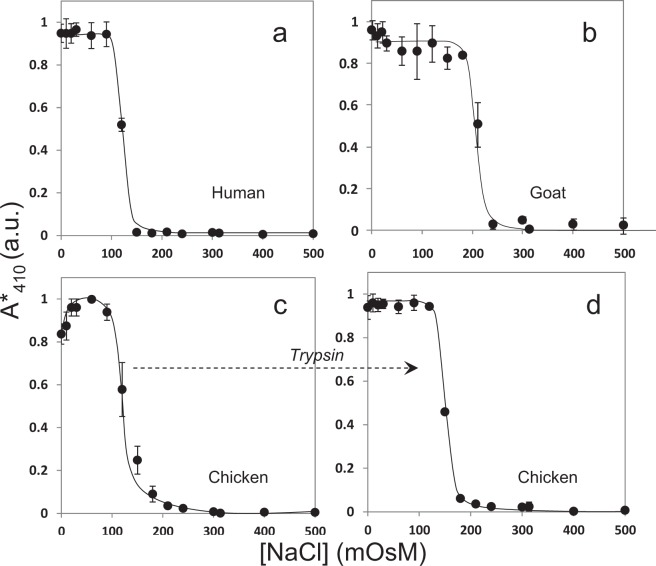
Fragiligrams obtained for (**a**) human, (**b**) goat, (**c**) chicken red blood cells (RBCs) and, (**d**) trypsinized-chicken RBCs using NaCl. Data shows fraction hemolysis measured as normalized absorbance (A* at 410 nm expressed in arbitrary units – see “Methods” for details) as a function of NaCl concentration (expressed in milli-osmolar units). All data shown is mean ± standard deviation (n = 3) from independent experiments.

Figure 1a,b show the fragiligrams obtained for human and goat RBCs in presence of 0 to 500 mOsM (i.e. 0 to 250 mM) sodium chloride (NaCl). As expected for mammalian RBCs, and in agreement with widely reported data in earlier literature, the fragiligrams are monotonic sigmoidal curves – with the highest release of hemoglobin in hypotonic solutions (starting with plain water, [NaCl] = 0 mOsM = 0 mM), which decreases with increase in osmolarity of the solutions.

### Non-monotonic osmotic fragility of avian RBCs in saline

Figure 1c shows the fragiligrams obtained for chicken RBC in the presence of 0 to 500 mOsM NaCl. Clearly, the fragiligrams are non-monotonic with maximal hemolysis around [NaCl] = 30 to 60 mOsM = 15 to 30 mM. While it was expected that the highest release of hemoglobin would be in a hypotonic solution with only water (i.e. [NaCl] = 0 mM = 0 mOsM), the data obtained was repeatedly different – the highest release of hemoglobin was observed at [NaCl] = 60 mOsM = 30 mM.

**Figure 2:**
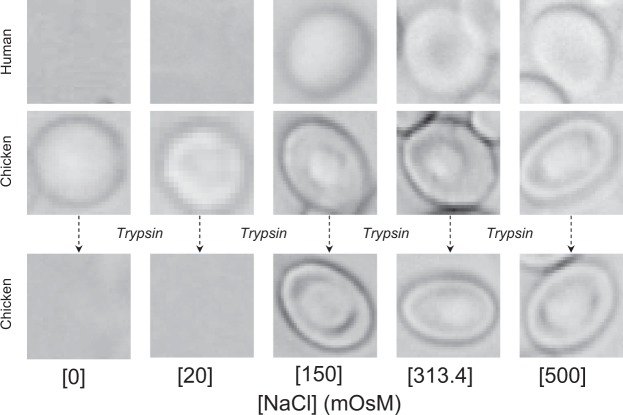
Representative images of human, chicken and, trypsinized-chicken RBCs at selected osmolarities of NaCl (see supplementary material for morphology of RBCs at all the different osmolarities of NaCl).

**Figure 3:**
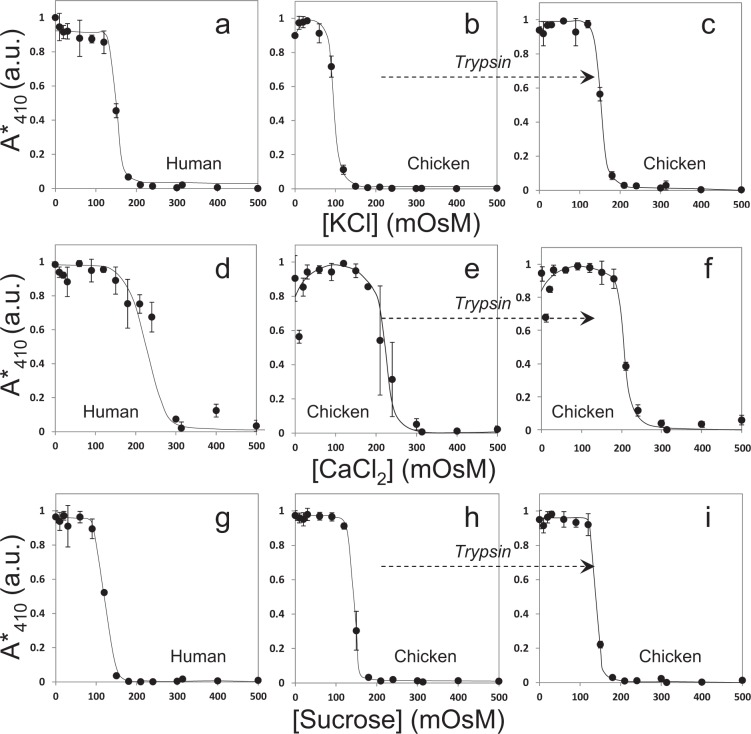
Fragiligrams obtained for (**a**) human RBCs, (**b**) chicken RBCs and, (**c**) trypsinized-chicken RBCs using KCl; (**d**) human RBCs, (**e**) chicken RBCs and, (**f**) trypsinized-chicken RBCs using CaCl_2_; (**g**) human RBCs, (**h**) chicken RBCs and, (**i**) trypsinized-chicken RBCs using sucrose. Data shows fraction hemolysis measured as normalized absorbance (A* at 410 nm expressed in arbitrary units – see “Methods” for details) as a function of osmolyte concentration (expressed in milli-osmolar units). All data shown is mean ± standard deviation (n = 3) from independent experiments.

The above interesting findings compelled us to carefully inspect the literature on osmotic behavior of avian RBCs in general. The important question was whether previous studies on avian RBCs were in agreement with our findings or not. Few previous studies have reported monotonic sigmoidal fragiligrams for avian RBCs^24,26,31^. However, in these studies, specific experimental protocols involving treatment of avian RBCs with mild hypotonic treatments for obtaining complete hemolysis have been developed and used^24,26,31^. For example, an additional step mild saline treatment (e.g. 0.22%) results in maximal hemolysis. Subsequently the data was normalized and presented in these studies by assuming the occurrence of this maximal hemolysis in the most hypotonic conditions. Even more interestingly, we found that non-monotonic fragiligrams of RBCs from different avian species have been repeatedly obtained in several studies. However, for some reason(s) the non-monotonic observations went unnoticed and/or were not explored further. For example, fragiligrams were reported for chicken^17^, duck^17^, pigeons^17^, Nigerian domestic fowls^23^, Hubbard fowls^23^, Guinea fowl^29^, Muscovy duck^29^ and Japanese quail^30^. Interestingly, all the fragiligrams are non-monotonic without exception and are in complete agreement with our discovery in this work. Proper inspection of the reported data (e.g. Figure 5 by Lewis and Fergusson^17^; Figs 1, 2 and 3 by Oyewale and Durotoye^23^; Figs 2 and 3 by Donaldson *et al*.^29^; Fig. 3 by Donaldson *et al*.^30^) clearly shows non-monotonic osmotic behavior of avian RBCs at hypotonic conditions with only up to ~80% hemolysis in plain water, and with maximal hemolysis at 0.1 to 0.25% sodium chloride corresponding to [NaCl] ~ 17 to 40 mM – these results are in full agreement with results shown in Fig. 1c of this work.

The above clearly shows that the non-monotonic osmotic behavior of chicken RBCs discovered in this work is applicable to avian RBCs in general.

### Conversion of the non-monotonic osmotic fragility of avian RBCs in saline to monotonic sigmoids

The non-monotonic osmotic behavior of avian RBCs is not explained by any mathematical model or mechanistic information on transport of water (or solutes) across biological membranes. Therefore, based on the reported presence of inactive/non-functional aquaporins on plasma membranes of chicken RBCs^41^, and the reported large influx of sodium ion current through sodium channels on plasma membranes of Xenopus oocytes subsequent to extracellular trypsin treatment^43^, we decided to treat the chicken RBCs with trypsin. The idea was to shave off the extracellular ectodomains of either aquaporins or sodium channels or both, along with other trypsin-sensitive ectodomains, in order to explore the possible regulation of sodium and water transport by any of trypsin-senstive ectodomains including the channels^15,42^. Figure 1d shows the fragiligrams obtained for trypsinized chicken RBCs. Clearly, the non-monotonic osmotic behavior of chicken RBCs had been converted by trypsin treatment to the expected monotonic sigmoidal form.

### Morphology of mammalian and avian RBCs in saline

Figure 2 shows bright field images of RBCs from humans and chicken, as well as trypsinized chicken RBCs, in solutions of different osmolarities. Note that while human RBCs do not have any nucleus and appear round, the chicken RBCs have a nucleus and appear ellipsoidal. Two key observations were made – (a) the morphologies of human and chicken RBCs under different conditions were as expected and as reported earlier^19^, and, (b) trypsinization does not affect the morphology of chicken RBCs. It is important to note from Fig. 1 that complete hemolysis of chicken RBCs is not observed at [NaCl] = 0 and 20 mOsM. The non-hemolyzed chicken RBCs show a swollen phenotype at [NaCl] ≤ 50 mOsM (similar to non-hemolyzed native human RBCs at [NaCl] = 150 mOsM). However, as observed for native human RBCs, trypsinized-chicken RBCs show complete hemolysis at the lowest concentrations of NaCl. Supplemental Figures S1 and S2 show images of complete fields of view from which individual RBCs are shown in Fig. 2. More fields of view with conditions shown in Fig. 1 are shown in Supplemental Figs S3, S4 and S5 to demonstrate that the images shown in Fig. 2 are indeed representative of general findings.

### Osmotic fragility of mammalian and avian RBCs using other osmolytes

Since the earlier results obtained were using different saline (with NaCl) solutions only, we decided to test the osmotic behavior of chicken RBCs using other osmolytes also, with human RBCs serving as controls. The expectation, as earlier, was to obtain monotonic sigmoidal fragiligrams while assuming that the non-monotonic osmotic behavior of chicken RBCs was saline (with NaCl) specific. Figure 3a–c show fragiligrams obtained for human RBCs, chicken RBCs and trypsinized-chicken RBCs using KCl as the osmolyte. The data is clearly similar to that as observed with NaCl. Fragiligrams of human RBCs is monotonous and the fragiligrams of chicken RBCs are non-monotonic which are converted to monotonic sigmoidal by controlled trypsinization of the cells.

Figure 3d–f show fragiligrams obtained for human RBCs, chicken RBCs and trypsinized-chicken RBCs using CaCl_2_ as the osmolyte. While the data for human RBCs was as expected, the data for chicken RBCs was interesting. Trypsinization of chicken RBCs did somewhat flatten the non-monotonicity of the fragiligrams at lower solute concentrations, but not as dramatically as in the case of sodium and potassium salts. Thus, in this case, trypsinization did not result in complete conversion of the non-monotonic data to monotonic sigmoidal. However, partial impact of trypsinization was clearly observed.

Our results clearly indicated that (a) trypsin treatment was not having as much effect on calcium transporting mechanisms in chicken RBCs (compared to sodium and potassium), (b) if trypsin treatment was affecting aquaporins on the chicken RBC surface, it was resulting in only partial “activation” of aquaporins since the conversion to completely monotonic sigmoidal osmotic behavior was not observed with calcium, and (c) shaving off extracellular ectodomains of sodium and potassium channels was resulting in loss of their activity for regulation of ion transport, but not their specificity (otherwise trypsin treatment in case of calcium as a solute would also result in similar results).

**Figure 4:**
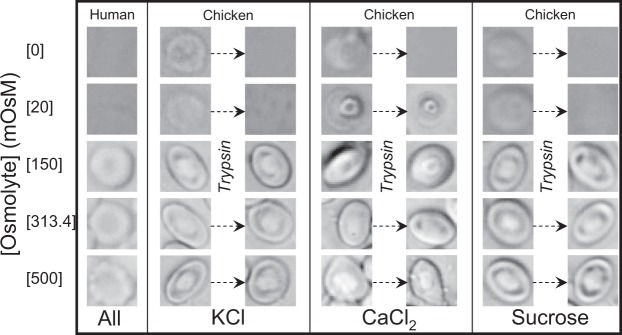
Representative images of human, chicken and, trypsinized-chicken RBCs at selected osmolarities of KCl, CaCl_2_, sucrose (see supplementary material for morphology of RBCs at all the different osmolarities of the osmolytes).

Finally, we used sucrose as a non-ionic osmolyte. Figure 3g–i show fragiligrams obtained for human RBCs, chicken RBCs and trypsinized-chicken RBCs while using sucrose as the osmolyte. Interestingly, the fragiligrams of chicken RBCs were monotonic sigmoidal in presence of sucrose but complete hemolysis was not observed by microscopy under any condition (Figs 4, S9 and S17). Further, fragiligrams of trypsinized-chicken RBCs was similar to non-trypsinized RBCs but complete hemolysis was observed by microscopy for trypsinized cells under hypotonic conditions (Figs 4, S9 and S18). It is possible that water uptake gets coupled with the uptake of sucrose as a nutrient resulting in “normal” appearing monotonic sigmoidal fragiligrams without trypsin treatment (i.e. in hypotonic conditions water is able to enter the cells along with sucrose resulting in osmotic release of hemoglobin but not osmotic bursting). With trypsin treatment, complete hemolysis was observed by microscopy in hypotonic conditions, with 3–3.5% higher maximum absorbance values post-trypsinization (however, see methods for details regarding comparison of absolute maximum absorbance values). This post-trypsinization complete hemolysis in hypotonic conditions again reflects trypsin-activation of aquaporins coupled with transport of water along with sucrose.

### Morphology of mammalian and avian RBCs using other osmolytes

Figure 4 shows bright field images of RBCs from humans and chicken, as well as trypsinized chicken RBCs, in solutions of different osmolarities for different osmolytes. As seen in Fig. 2, while human RBCs do not have any nucleus and appear round, the chicken RBCs have a nucleus and appear ellipsoidal. However, at hypotonic concentrations of CaCl_2_, the morphology of chicken RBCs is very different and only the nucleus seems to be predominantly visible. Further, the two key observations (a) and (b) for Fig. 2 remained the same here also. It is important to note from Fig. 3 that complete hemolysis of chicken RBCs is not observed at the lowest concentrations of any osmolyte. The non-hemolyzed chicken RBCs show a swollen phenotype at [Osmolyte] = 0 mOsM (similar to non-hemolyzed native human RBCs at [Osmolyte] = 150 mOsM). However, as observed for native human RBCs, trypsinized-chicken RBCs show complete hemolysis at the lowest concentrations of the osmolytes. Supplemental Figures S6, S7, S8 and S9 show images of complete fields of view from which individual RBCs are shown in Fig. 4. More fields of view with conditions shown in Fig. 4 are shown in Supplemental Figs S10–S18 to demonstrate that the images shown in Fig. 4 are indeed representative of general findings.

### Measurement of osmotic fragility of mammalian and avian RBCs using different osmolytes at different wavelengths

The absorption spectrum of hemoglobin from RBCs (both mammalian and chicken) shows a strong peak at 410 nm and another small peak at 540 nm (data confirmed by us and not shown since it has been published extensively including the literature cited in this work). Fragiligrams have been obtained in literature at both 410 nm and 540 nm. Therefore, while Figs 1 and 3 show the fragiligrams at 410 nm, we acquired the data at 540 nm also. Supplemental Figures S19, S20 and S21 show the fragiligrams for all the experiments at 410 nm and 540 nm. It is clear that the wavelength does not affect any of our main findings in this study, especially the non-monotonic osmotic behavior of chicken RBCs in presence of ionic osmolytes and the conversion of this behavior to monotonic sigmoidal in case of sodium and potassium salts by trypsin treatment.

## Discussion

Here, we would like to note that several more interpretations are possible by careful observation of the experimental data (i.e. fragiligrams and morphological characteristics). However, the discovery of non-monotonic osmotic behavior of chicken RBCs using ionic osmolytes is of primary importance in this work. It opens several exciting avenues for further detailed studies on expression of different channels and/or pumps coupled with proper morphological characterizations of avian erythrocytes from different species. These studies would be significant from the perspective of not only avian physiology but also towards gaining mechanistic insights into varying transport phenomena across biological membranes in general.

An interesting aspect is that earlier reports on comparisons of RBCs from amphibians, reptiles, birds (represented by domestic chicken – as in our case) and mammals do find “lower osmotic fragility” for birds^26^. This lower fragility (i.e. resistance to osmotic stress and robustness) is attributed primarily to their ellipsoidal shapes along with presence of organelles such as nucleus and mitochondria^18,19,26^, without any logical or mechanistic reason. We, on the other hand, provide a direct experimental measure of the “lower osmotic fragility” of avian RBCs in terms of non-monotonic fragiligrams. In addition, we show the possibility of increasing water transport by activation of non-functional aquaporins and/or regulation of specific activity of ion channels and/or cleavage of trypsin-sensitive ectodomains of plasma membrane proteins in avian RBCs. Of course, future experiments on direct measurements on activities of channels would be required to confirm the above possibility.

The findings reported in this work, while possibly having profound implications in our understanding of evolution of vertebrates, may have a direct impact on our understanding of avian physiology. For example, one direct physiological impact of hypotonic “burst-resistance” of avian erythrocytes to pure water (i.e. [Solute] = 0 mOsM = 0 mM) discovered in this work may be related to the ability of some species of birds to withstand long migratory flights while utilizing only salt-free moisture/water-vapor at high altitudes. Mammals, on the other hand, suffer from blood thinning and erythrocyte lysis (amongst other complications) if only salt-free water is provided for long periods of time. It is interesting to note that fish, amphibians and mammals evolved in salt water and/or in presence of water that always had some solutes/osmolytes dissolved in terrestrial conditions and never encountered any solute/osmolyte free source of water. Other evolutionary advantages to birds for having “burst-resistant” erythrocytes in pure water, or evolutionary reasons for mammalian erythrocytes losing the “burst-resistance” in pure water may be explored in future studies.

Finally, our findings provide anecdotal, but strong, evidence towards an interesting aspect of avian (especially chicken) physiology. It is often observed that poultry farms serving as meat sources often have anemic birds compared to wild or free-range birds. One clear reason for this could be high salt diet fed to animals in captivity resulting in “salt-based osmotic lysis” of their RBCs – while “salt-based osmotic lysis” is counter-intuitive in terms of literature, our findings clearly prove it. This “salt-based osmotic lysis” may also provide a possible explanation for reports emerging once-in-a-while on sudden deaths of flocks of birds in some locations across the globe.

## Methods

Chicken whole blood was collected from local chicken-meat shops (in the Hauz Khas, Kalu Sarai areas of New Delhi, India). Goat whole blood was collected from slaughter houses in Ghaziabad, Uttar Pradesh, India – however due to social difficulties in gaining access to slaughter houses, limited experiments were performed with goat RBCs. Isolation of RBCs from blood samples was done at Indian Institute of Technology Delhi (IIT Delhi) as per the guidelines and regulations of the IIT Delhi biosafety committee. The experimentation did not involve any human or animal subjects directly. Human whole blood donated by healthy individuals to National Institute of Pathology (Indian Council of Medical Research – ICMR, Safdarjung Hospital Campus, New Delhi, India) was used, as per approval from National Institute of Pathology biosafety committee and in accordance with ICMR guidelines and regulations (including obtaining of informed consent from donors), for isolating RBCs. For all samples (human, goat and chicken), whole blood was collected in polypropylene tubes without adding any anticoagulant, and RBCs were separated from the blood plasma to avoid interference of any other blood plasma components with our experimental assays. First, RBCs were separated from whole blood by suspending in PBS (1X, pH 7.4) and by spinning at 650 g for 5 minutes at 4 °C using an Eppendorf (India) benchtop 5810R centrifuge. Again after centrifugation pelleted RBCs were resuspended in PBS (1X, pH 7.4) and centrifuged at 650 g for 5 minutes at 4 °C – this process was repeated thrice with phosphate buffered saline (pH 7.4). Counting of red blood cells (isolated from chicken, goat and human blood) was done by using Haemocytometer under upright Motic Biological Microscope. Final concentration of RBCs used for further experiments was 5 × 10^5^ cells/μl. RBCs were suspended in osmolyte solutions of solutes mixed in Milli Q water. RBCs suspended in PBS (1X, pH 7.4) were also observed. NaCl, KCl, CaCl_2_.2H_2_O, sucrose and all other analytical grade reagents were obtained from Merck and Fisher Scientific, India. Microtiter plates (BD Biosciences India) were used for spectrophotometric measurements made with Multiskan-GO-microplate spectrophotometer from Thermo Scientific, India.

### Preparation of chicken RBCs without trypsin treatment and with trypsin treatment

After isolating RBCs from whole blood and suspending in PBS (1X, pH 7.4) 4 °C, the RBCs were divided equally into two centrifuge tubes. First tube of suspended RBCs was incubated at room temperature (~25 °C) for 30 minutes. Second tube of RBCs was treated by addition of trypsin (0.2 mg/ml in PBS 1X, pH 7.4) followed by a 30 minute incubation at room temperature. After incubating at room temperature for 30 minutes, the first tube (RBCs without trypsin treatment) was centrifuged at 1500 g for 10 minutes and the pellet of RBCs was washed twice with PBS. In the second tube (having RBCs and trypsin), trypsin inhibitor (0.4 mg/ml in PBS 1X, pH 7.4) was added – this tube was incubated again at room temperatue for five minutes and then centrifuged at 1500 g for 10 minutes followed by washing the pellet of trypsinized RBCs twice in PBS.

### Osmotic Fragility (OF) measurements of RBCs in varying concentrations of osmolytes

Different osmolarity solutions of NaCl, KCl, CaCl_2_.2H_2_O and sucrose, ranging from 10mOsM to 500 mOsM were prepared in Milli Q water. RBCs suspended in Milli Q water and PBS (1X, pH 7.4) were expected to serve as positive and negative controls (for hemolysis) respectively in each set for both chicken and human RBCs – however due to the results, hemolysis in water did not serve as a positive control with chicken RBCs (this was addressed as explained below). 40 µl RBCs (suspended in PBS as mentioned above) were thoroughly mixed in 1 ml osmolyte solutions by gentle pipetting. Samples were kept for incubation for 1 hour at room temperature. After incubation, samples were spun at 650 g for 5 minutes at 4 °C to pellet out non-hemolysed RBCs. 100 μl of supernatant containing hemoglobin from each tube was transferred to 96-well microtiter plates. Absorbance of the samples was then read at 410 nm and 540 nm on the Multiskan-GO plate-reader. For preparing fragiligrams, osmotic fragility data was measured on a scale of 0 to 1.0 as normalized absorbance A* = (A − A_Min_)**/**(A_Max_ − A_Min_), where A_Max_ was the maximum absorbance and A_Min_ was the minimum absorbance, at 410 nm and 540 nm respectively. Here it is pertinent to mention that the raw value of A_Max_ obtained in the initial experiments were confirmed to be similar (almost same as) to values obtained by lysing the RBC suspensions with 1% Triton-X100 and 0.5% SDS solutions which served as positive controls for hemolysis along with microscopic observation of RBC suspensions in all experiments (absence of any intact cells also indicated complete hemolysis). It is also important to note that absolute maximum absorbance values are not compared during osmotic fragility measurements due to (a) sensitivity of absorbance to oxidation state of hemoglobin^16^, (b) sensitivity of absorbance values to minor fluctuations in the electrical supply to the spectrophotometer, and (c) possible variations in the amounts/yields and absorbance of hemoglobin in different RBCs isolated from different blood samples.

### Imaging and image acquisition

Morphology of RBCs were observed using the bright field of inverted Olympus Microscope IX51 (Olympus, Japan) and images were acquired using Olympus CCD camera DP70 (Olympus, Japan) attached to the microscope.

### Data analysis

All data are expressed as the mean ± standard deviation for representative triplicates (n = 3). The data were analyzed and plotted using Microsoft Excel.

## Supplementary information

Supplementary Information
