# Supplementary material for: Osmotic tolerance of avian erythrocytes to complete hemolysis in solute free water
Source: Sci Rep. 2019 May 28;9:7976. doi: 10.1038/s41598-019-44487-7 (PMC6538707; doi:10.1038/s41598-019-44487-7)
Supplement: Supplementary file 1 — Supplementary Information [file 41598_2019_44487_MOESM1_ESM.pdf]

Osmotic tolerance of avian erythrocytes to complete hemolysis in solute free water

Snigdha Singh, Nisha Ponnappan, Anand Verma & Aditya Mittal

Supplementary Figures S1 to S21

Osmolyte Concentration (mM)

[0]

[20]

[150]

[313.4]

[500]

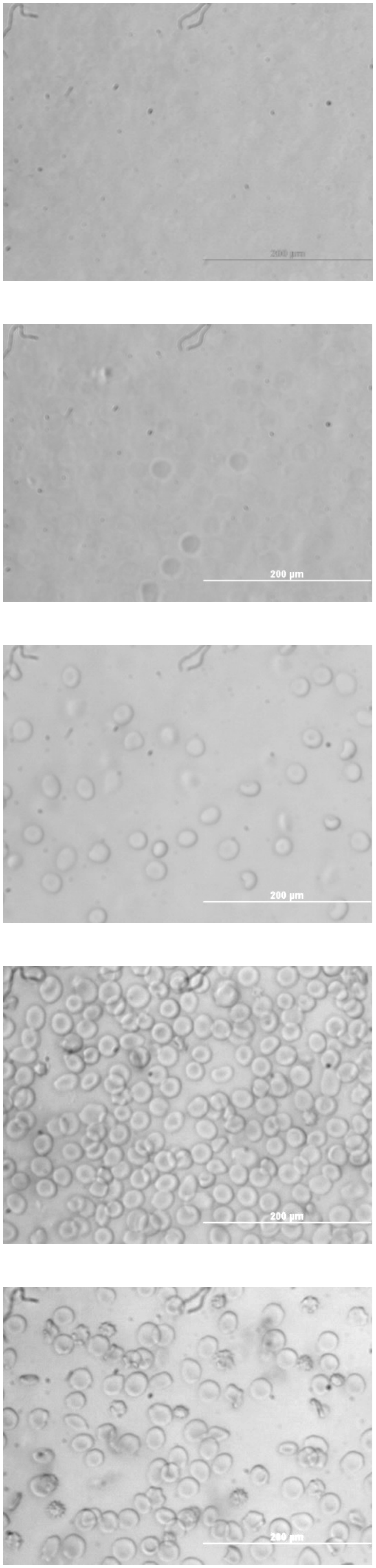

Figure S1: Human RBCs

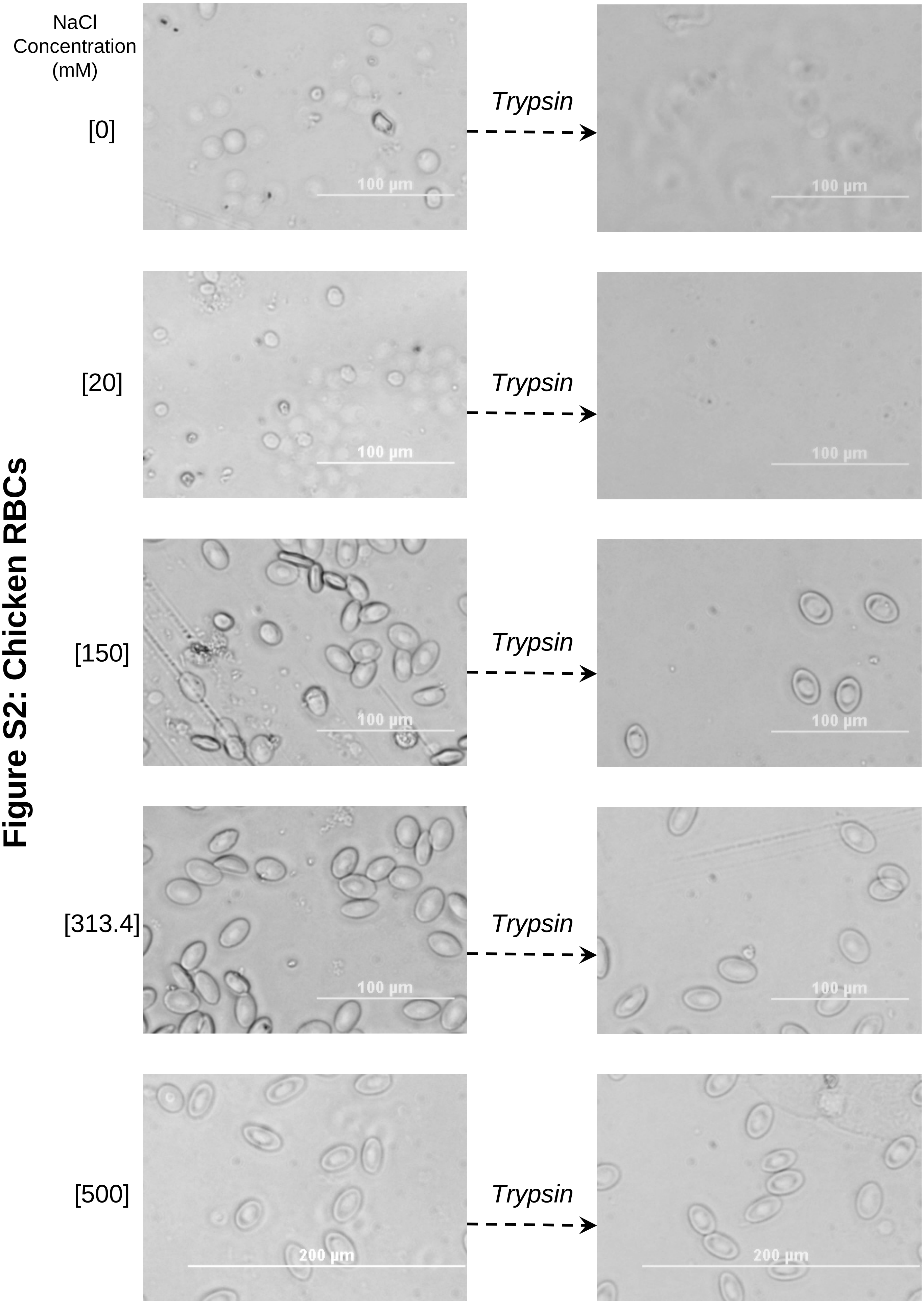

Figure S2: Chicken RBCs

Figure S3: Human RBCs-NaCl

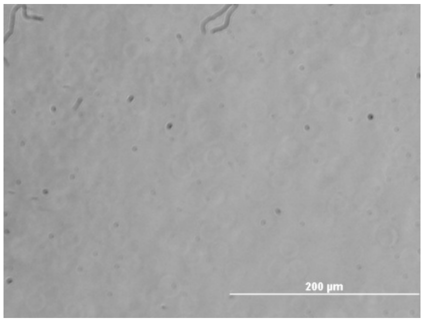

0mOsM

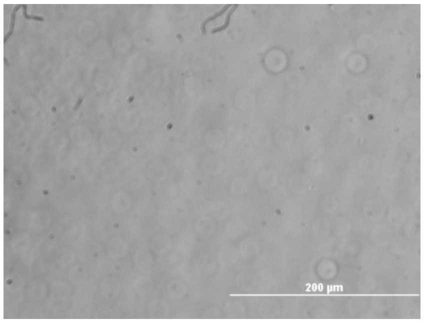

10mOsM

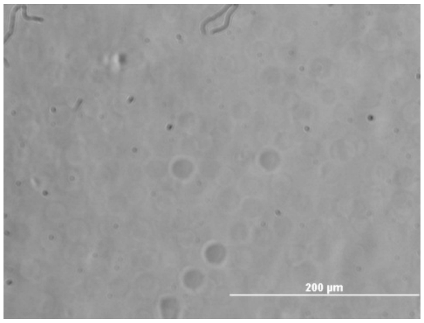

20mOsM

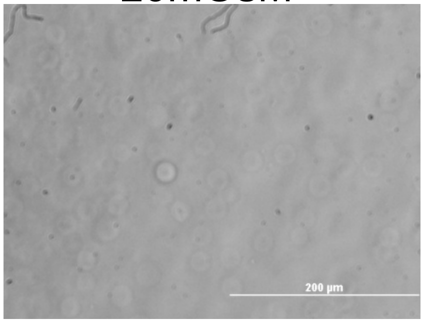

30mOsM

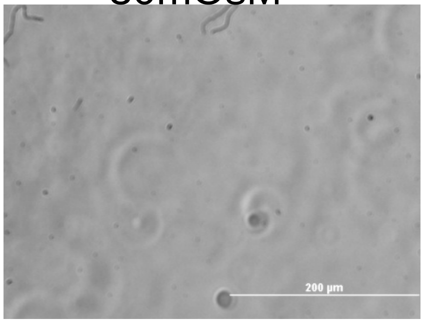

90mOsM

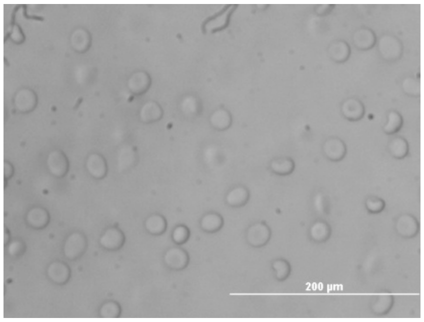

150mOsM

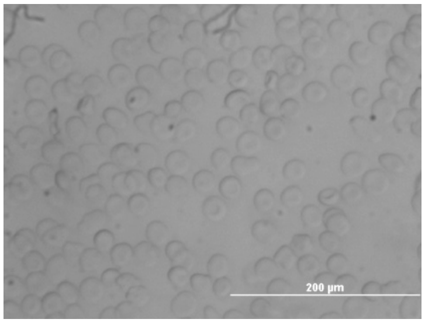

210mOsM

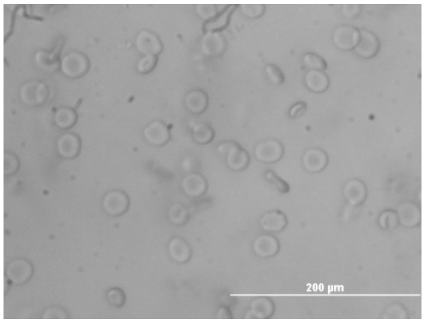

300mOsM

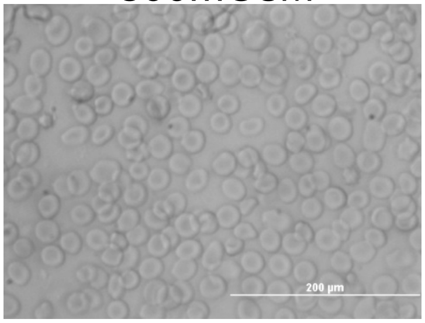

313.4mOsM

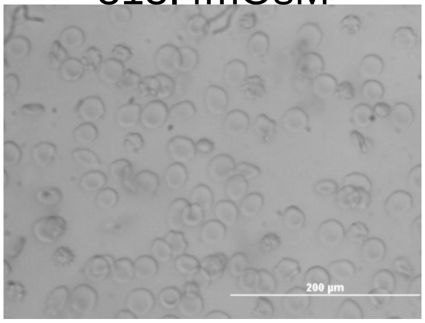

500mOsM

Figure S4: Chicken RBCs(Without Trypsin Treatment)-NaCl

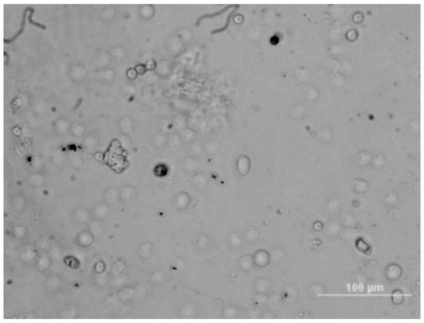

0mOsM

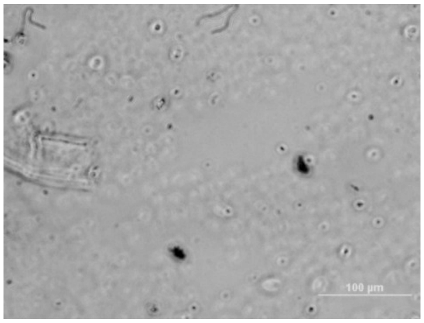

10mOsM

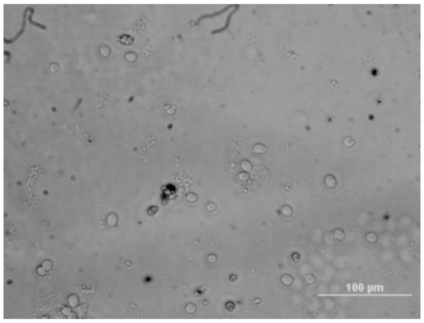

20mOsM

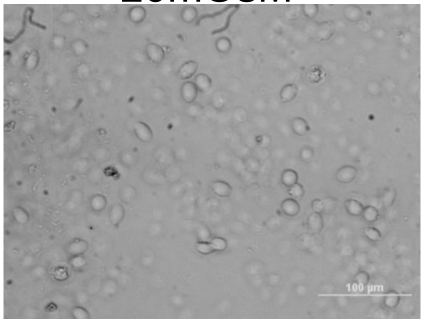

30mOsM

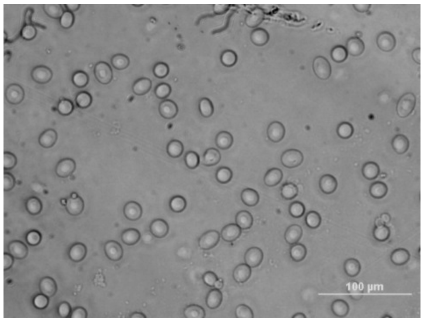

90mOsM

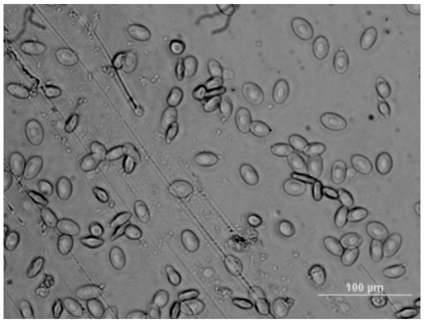

150mOsM

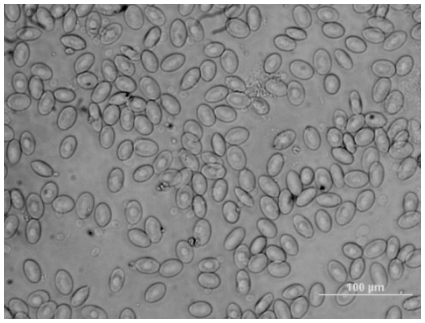

210mOsM

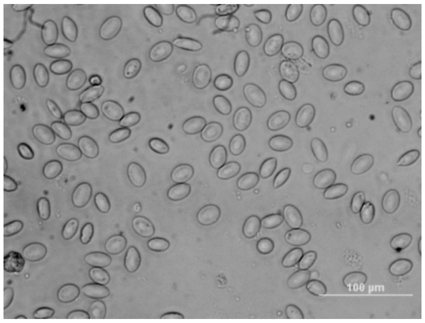

300mOsM

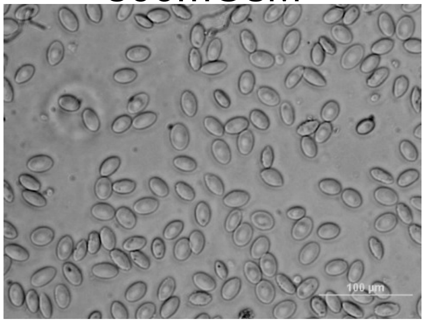

313.4mOsM

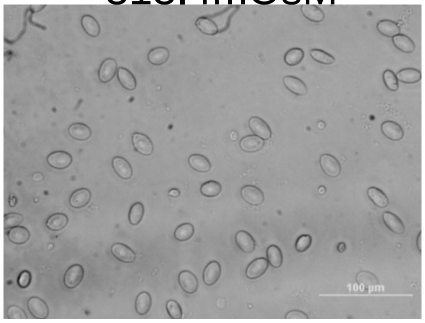

5000mOsM

Figure S5: Chicken RBCs(With Trypsin Treatment)-NaCl

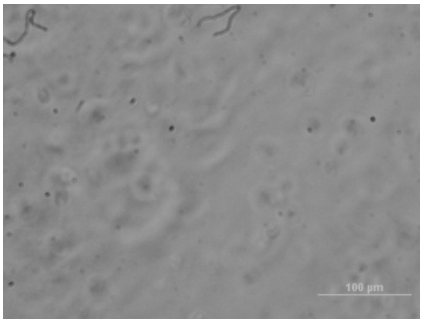

0mOsM

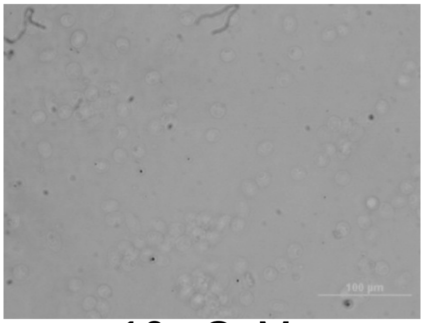

10mOsM

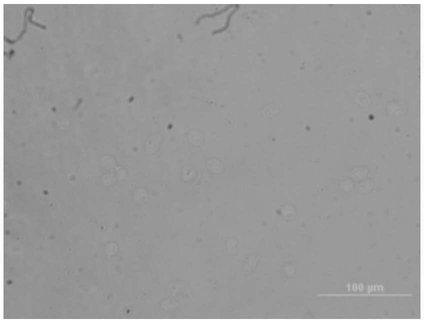

20mOsM

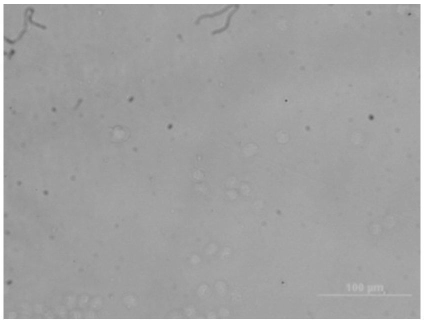

30mOsM

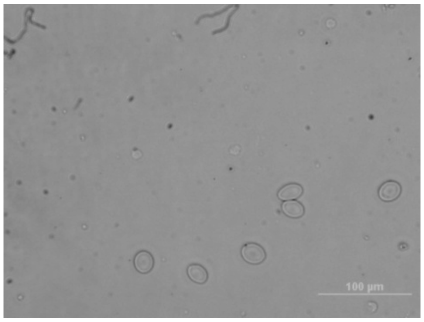

90mOsM

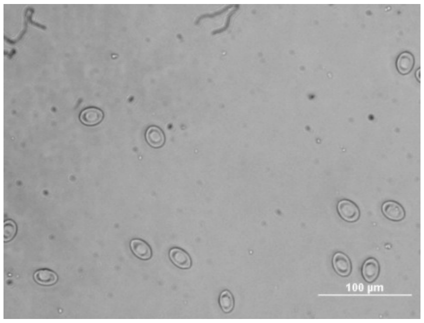

150mOsM

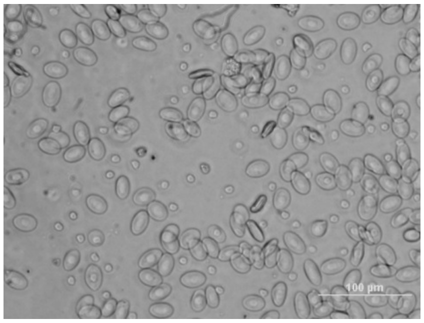

210mOsM

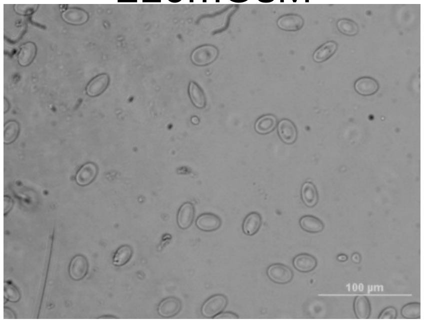

300mOsM

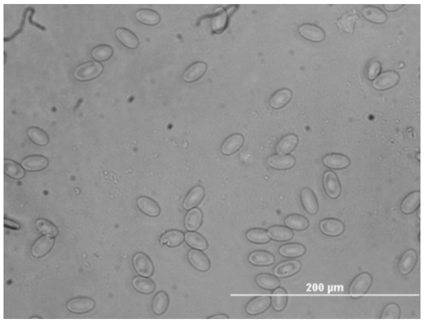

313.4mOsM

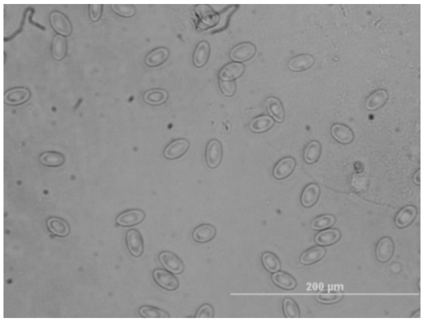

500mOsM

Figure S6: Human RBCs

Osmolyte Concentration (mM)

[0]

[20]

[150]

[313.4]

[500]

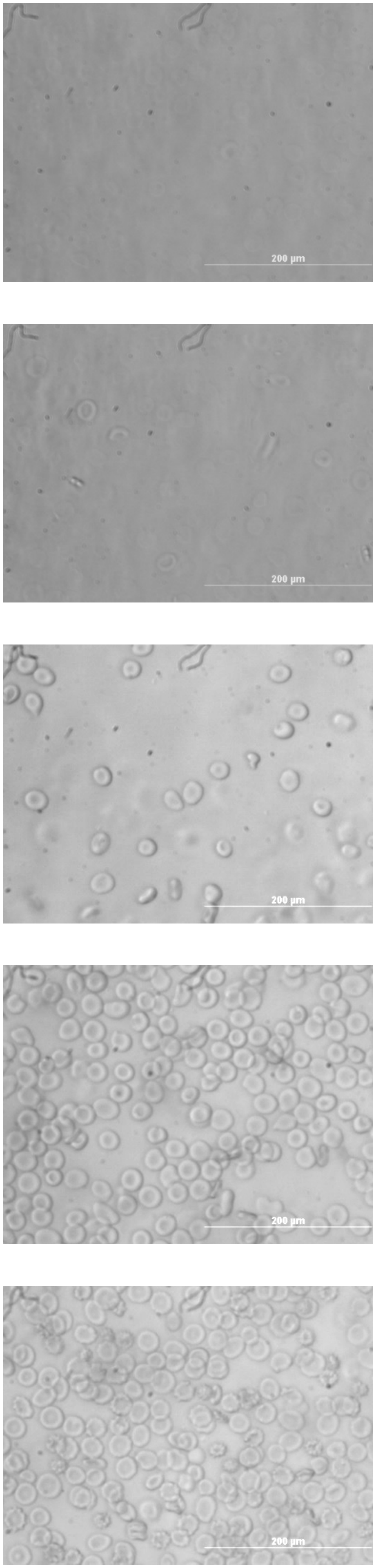

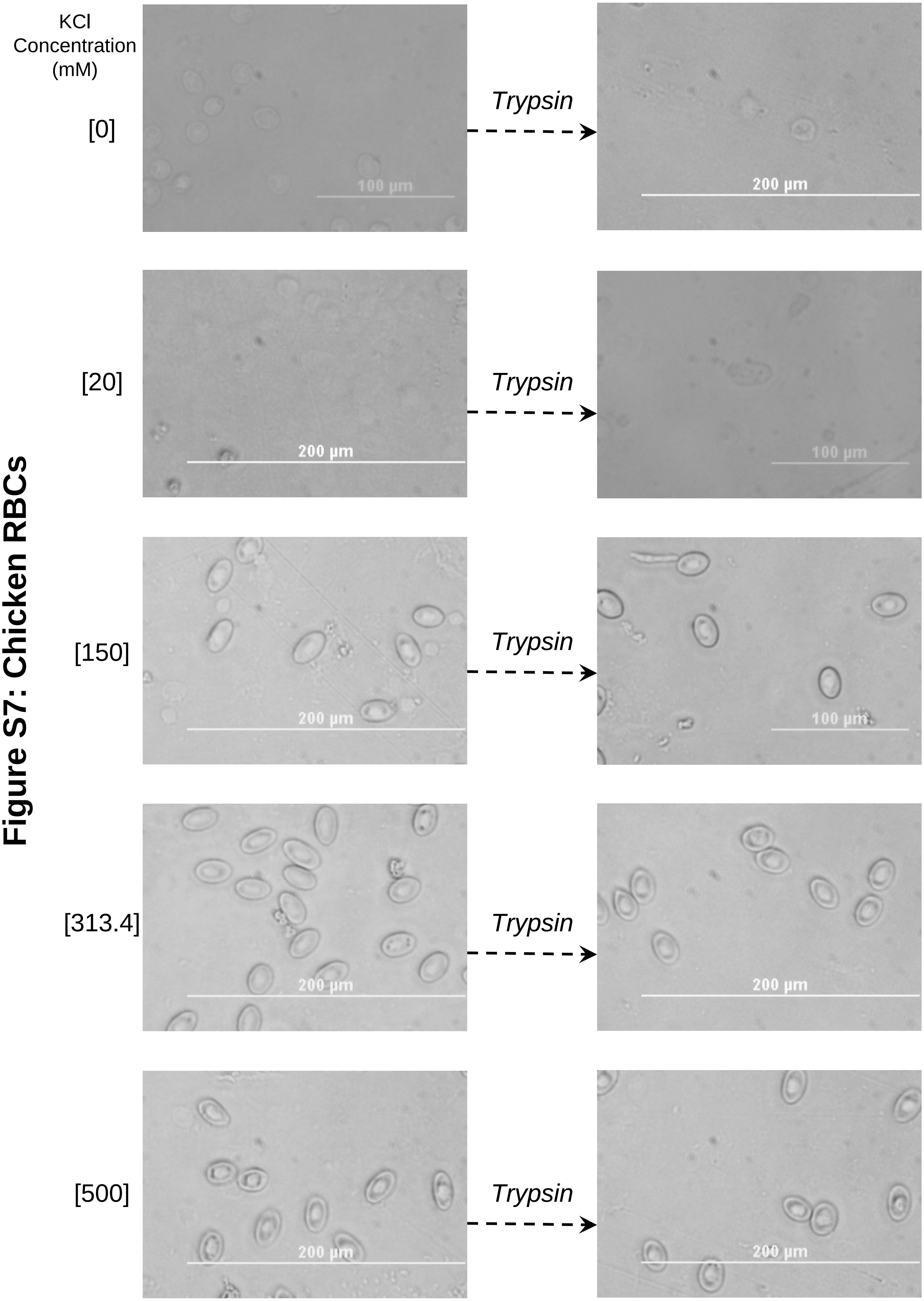

Figure S7: Chicken RBCs

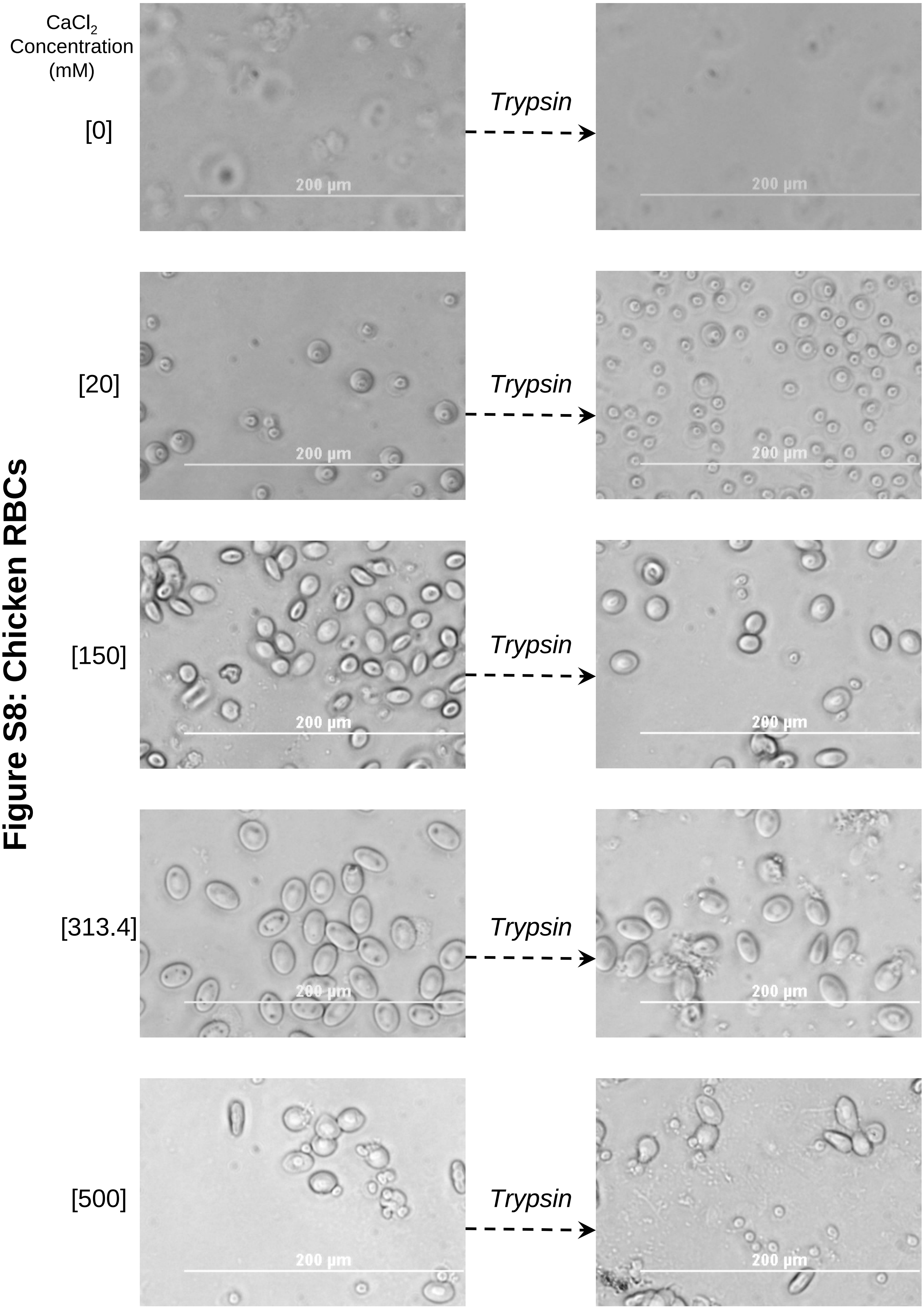

Figure S8: Chicken RBCs

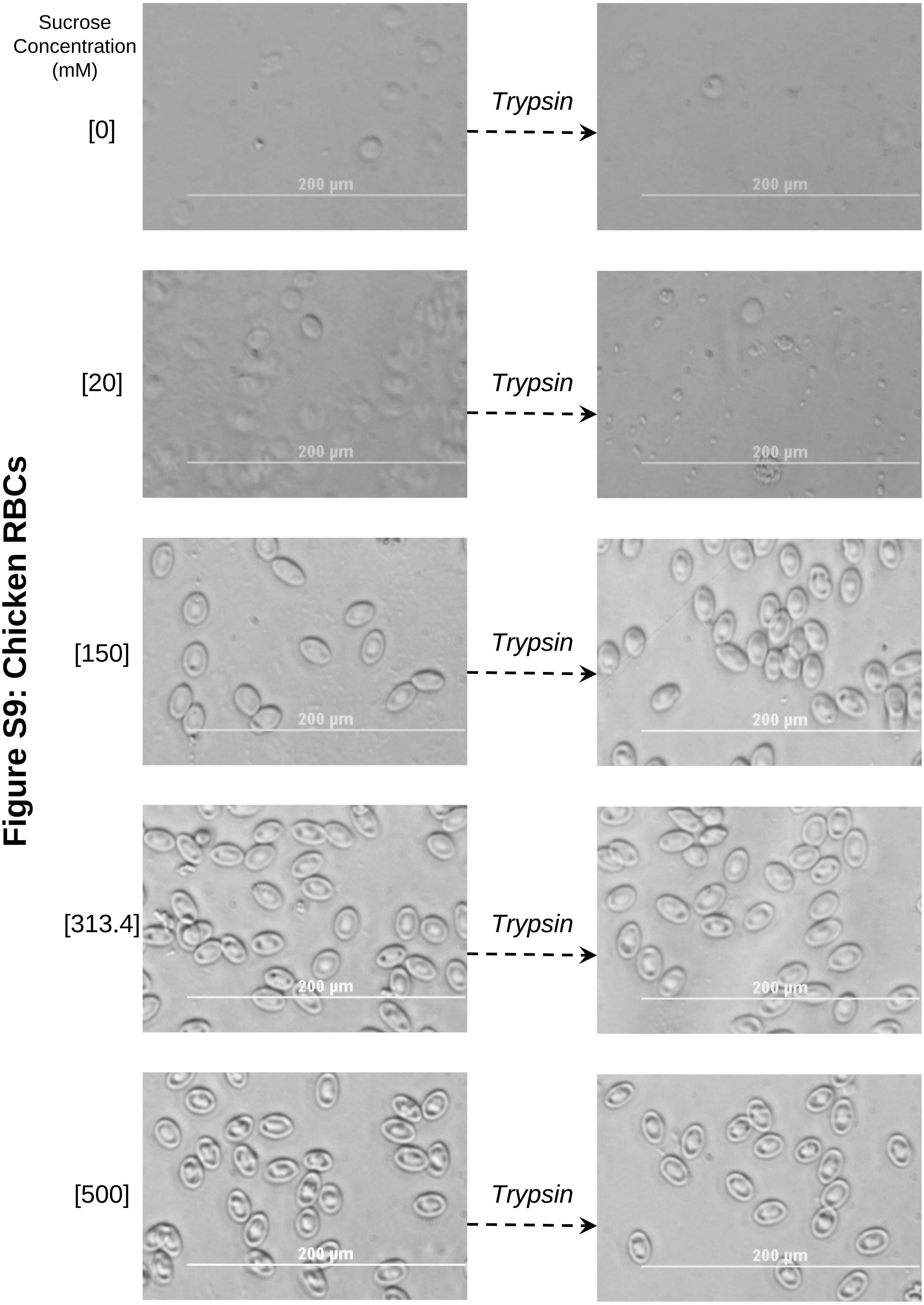

Figure S9: Chicken RBCs

Figure S10: Human RBCs-KCl

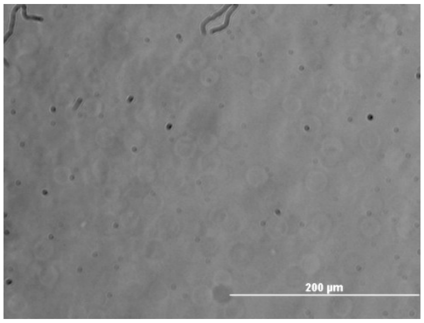

0mOsM

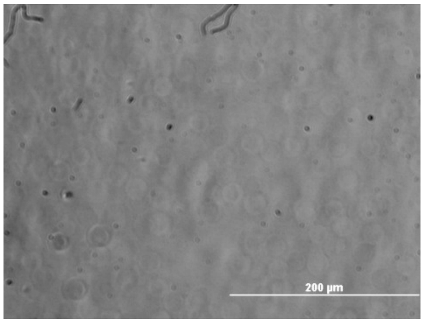

10mOsM

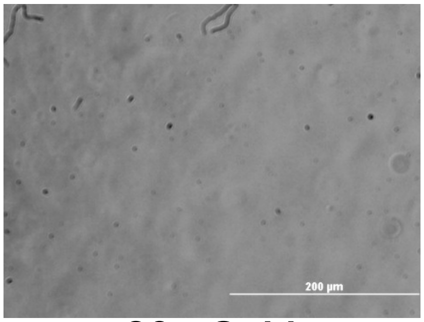

20mOsM

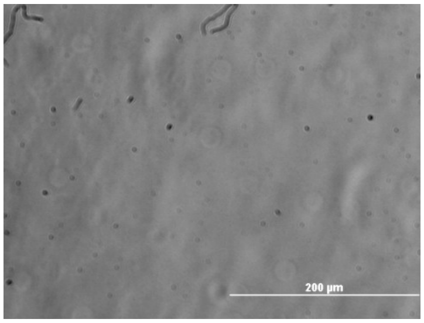

30mOsM

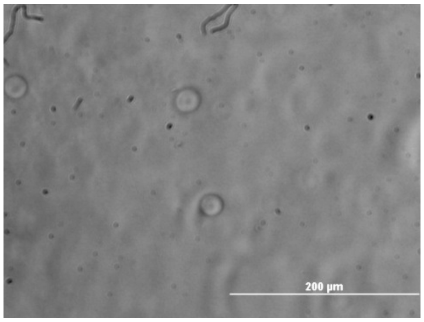

90mOsM

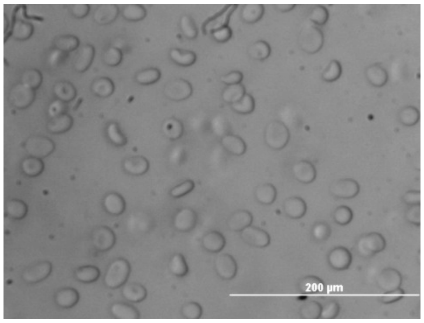

150mOsM

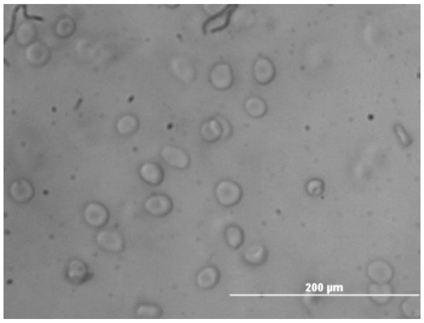

210mOsM

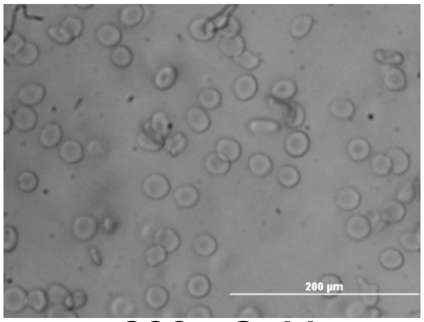

300mOsM

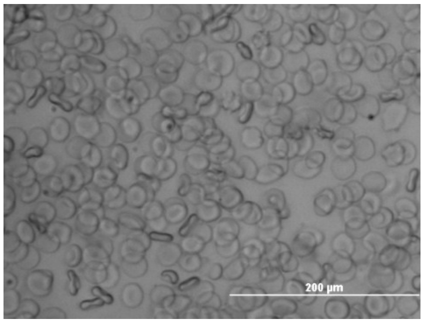

313.4mOsM

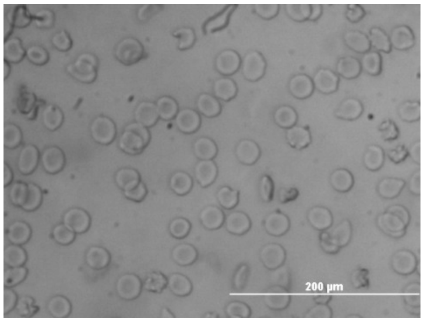

500mOsM

Figure S11: Chicken RBCs(Without Trypsin Treatment)-KCl

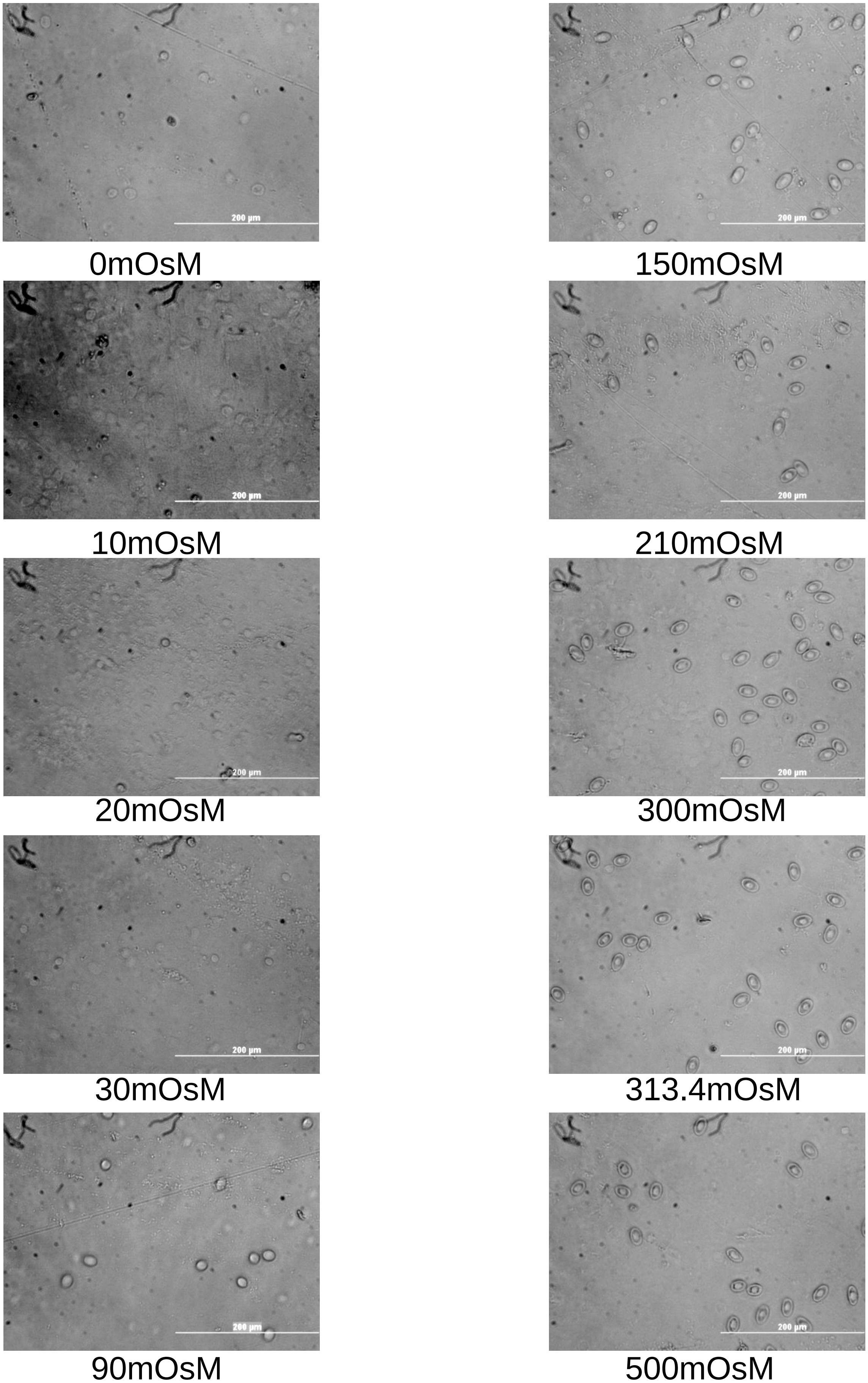

Figure S12: Chicken RBCs(WithTrypsin Treatment)-KCl

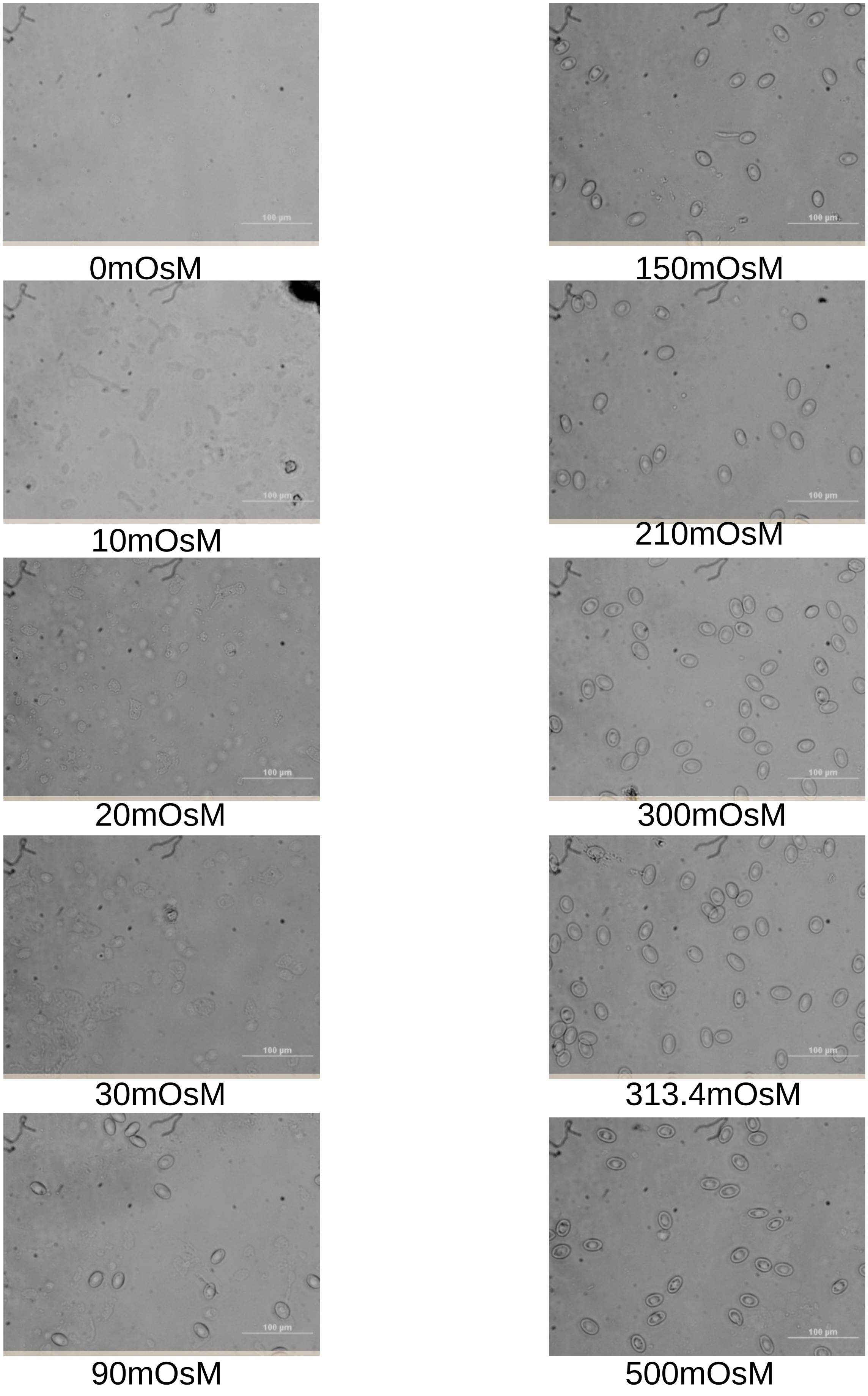

Figure S13: Human RBCs-$CaCl_2$

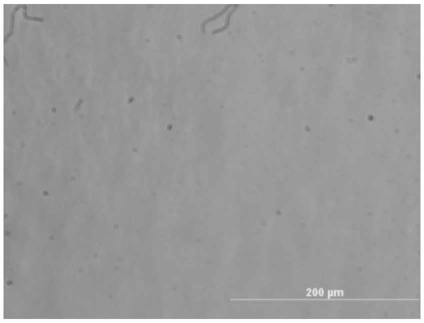

0mOsM

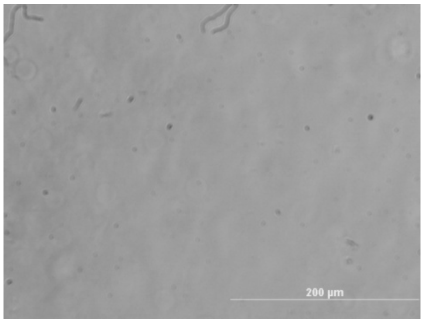

10mOsM

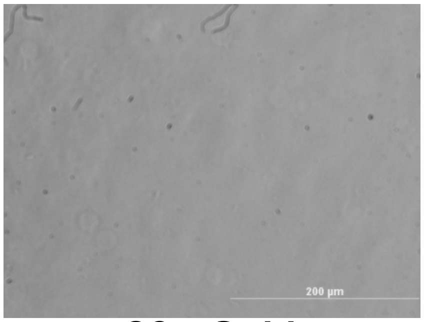

20mOsM

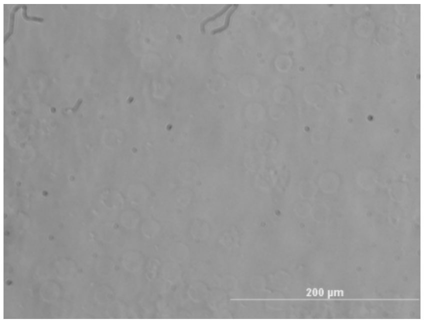

30mOsM

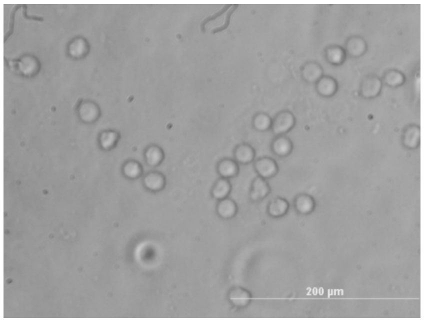

90mOsM

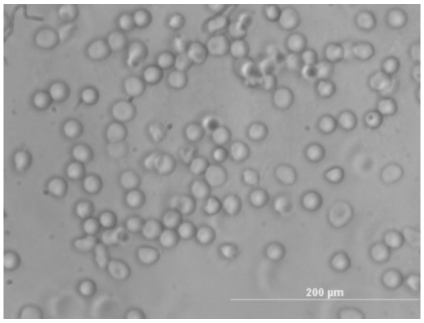

150mOsM

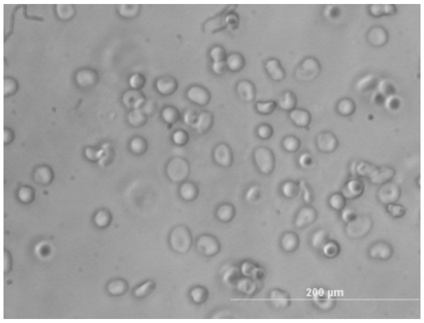

210mOsM

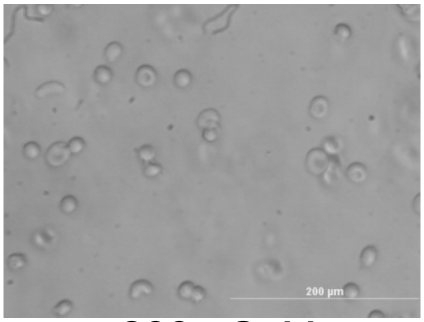

300mOsM

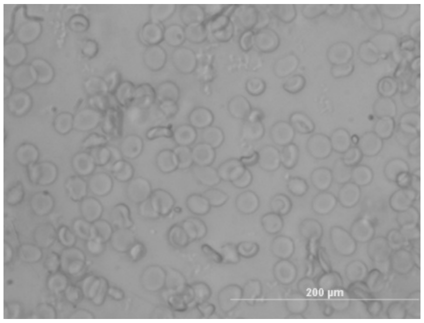

313.4mOsM

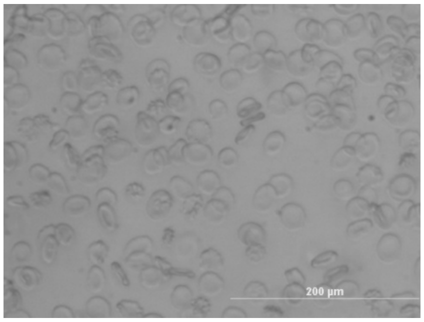

500mOsM

Figure S14: Chicken RBCs(Without Trypsin Treatment)-$CaCl_2$

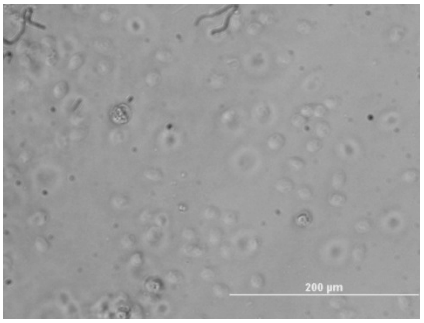

0mOsM

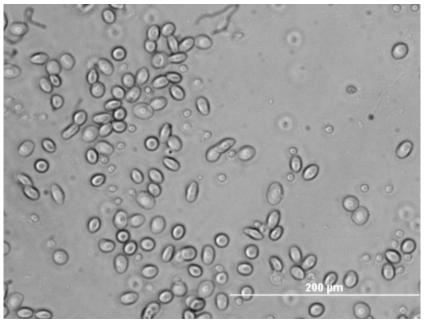

150mOsM

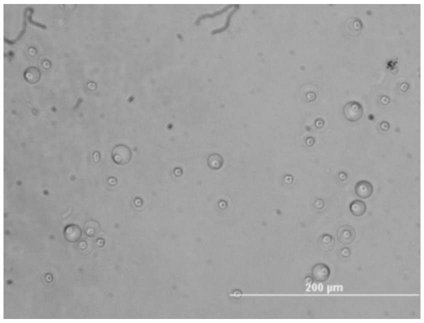

10mOsM

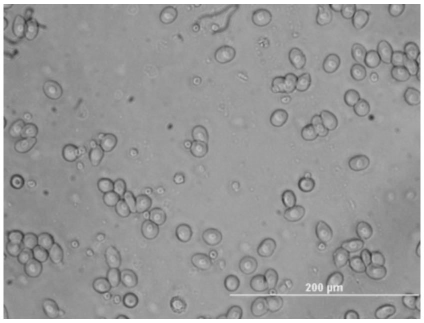

210mOsM

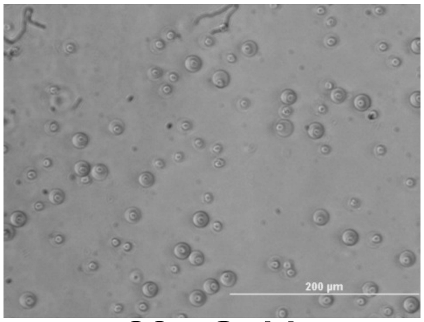

20mOsM

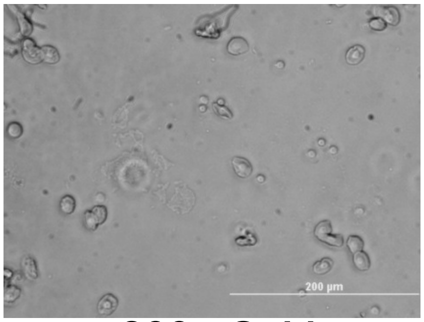

300mOsM

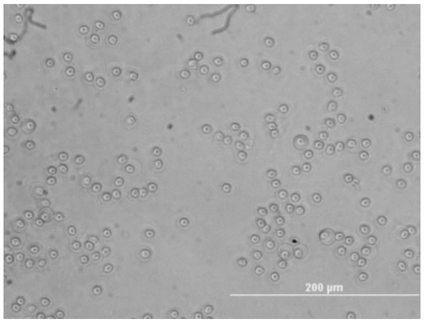

30mOsM

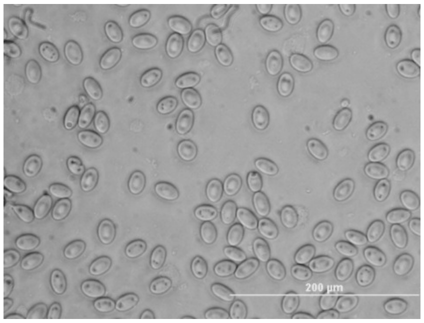

313.4mOsM

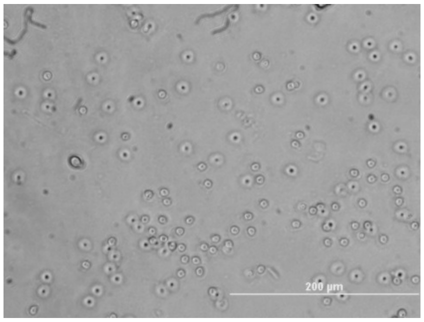

90mOsM

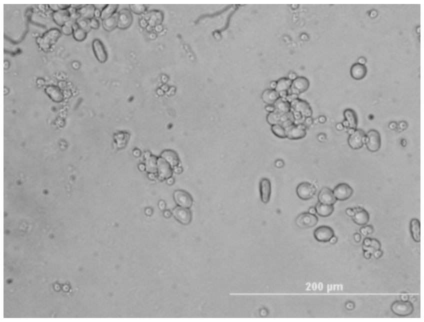

500mOsM

Figure S15: Chicken RBCs(With Trypsin Treatment)-$CaCl_2$

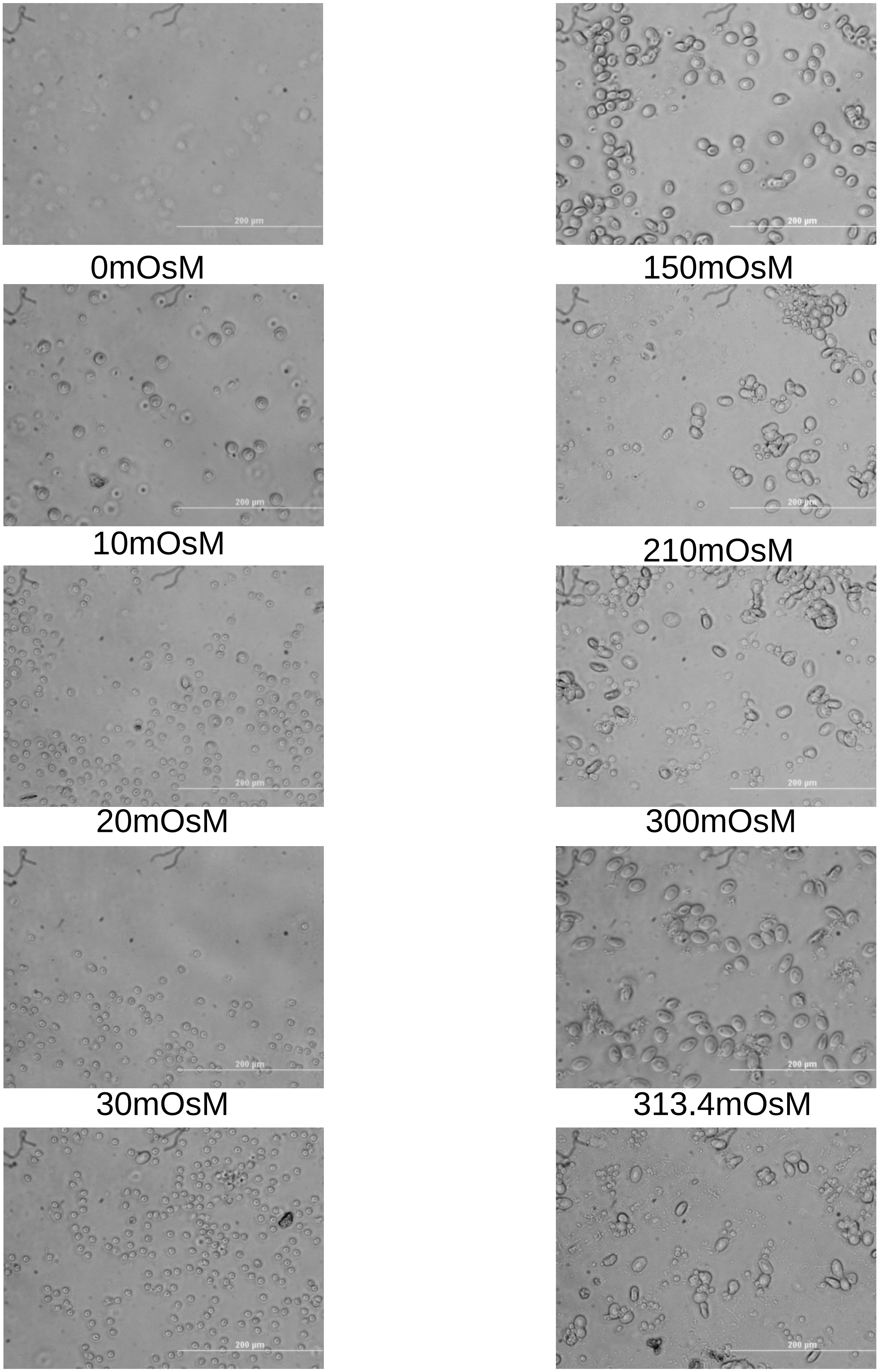

0mOsM 150mOsM

10mOsM 210mOsM

20mOsM 300mOsM

30mOsM 313.4mOsM

90mOsM 500mOsM

Figure S16: Human RBCs-Sucrose

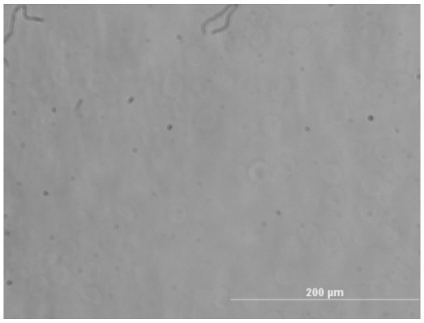

0mOsM

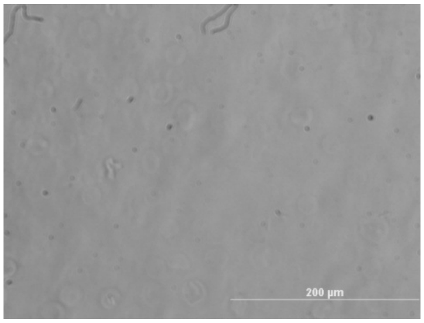

10mOsM

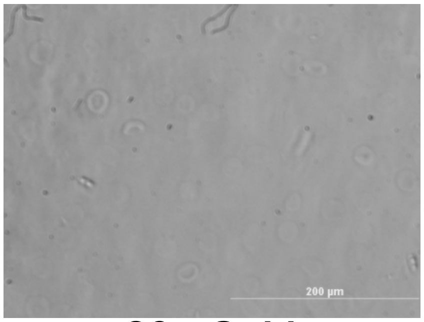

20mOsM

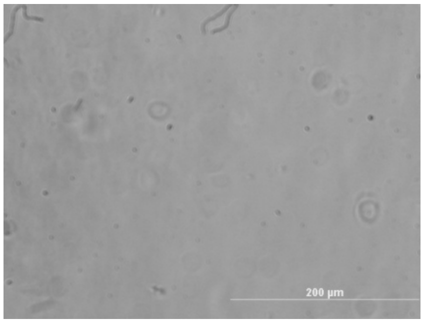

30mOsM

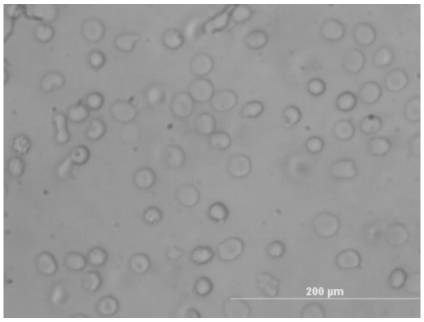

90mOsM

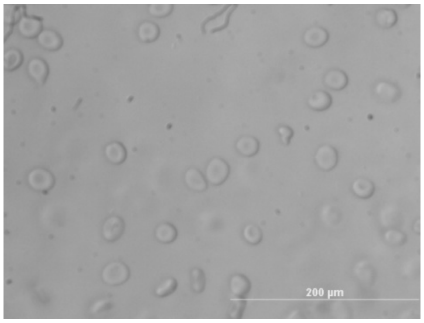

150mOsM

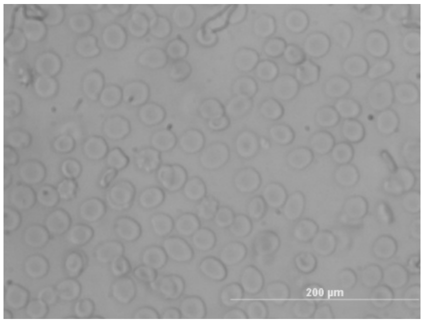

210mOsM

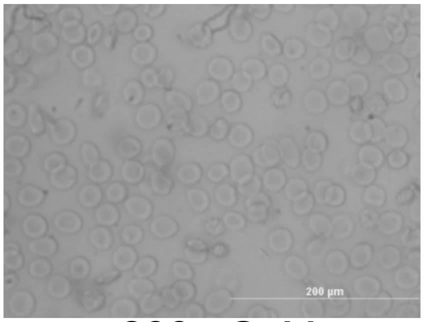

300mOsM

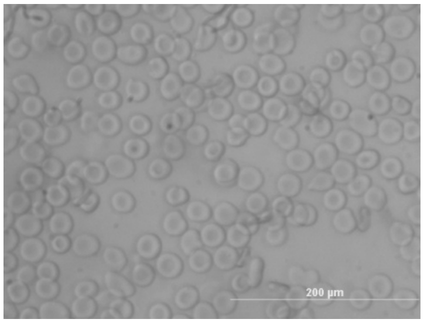

313.4mOsM

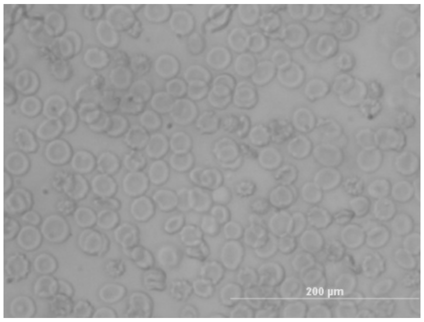

500mOsM

Figure S17: Chicken RBCs(Without Trypsin Treatment)-Sucrose

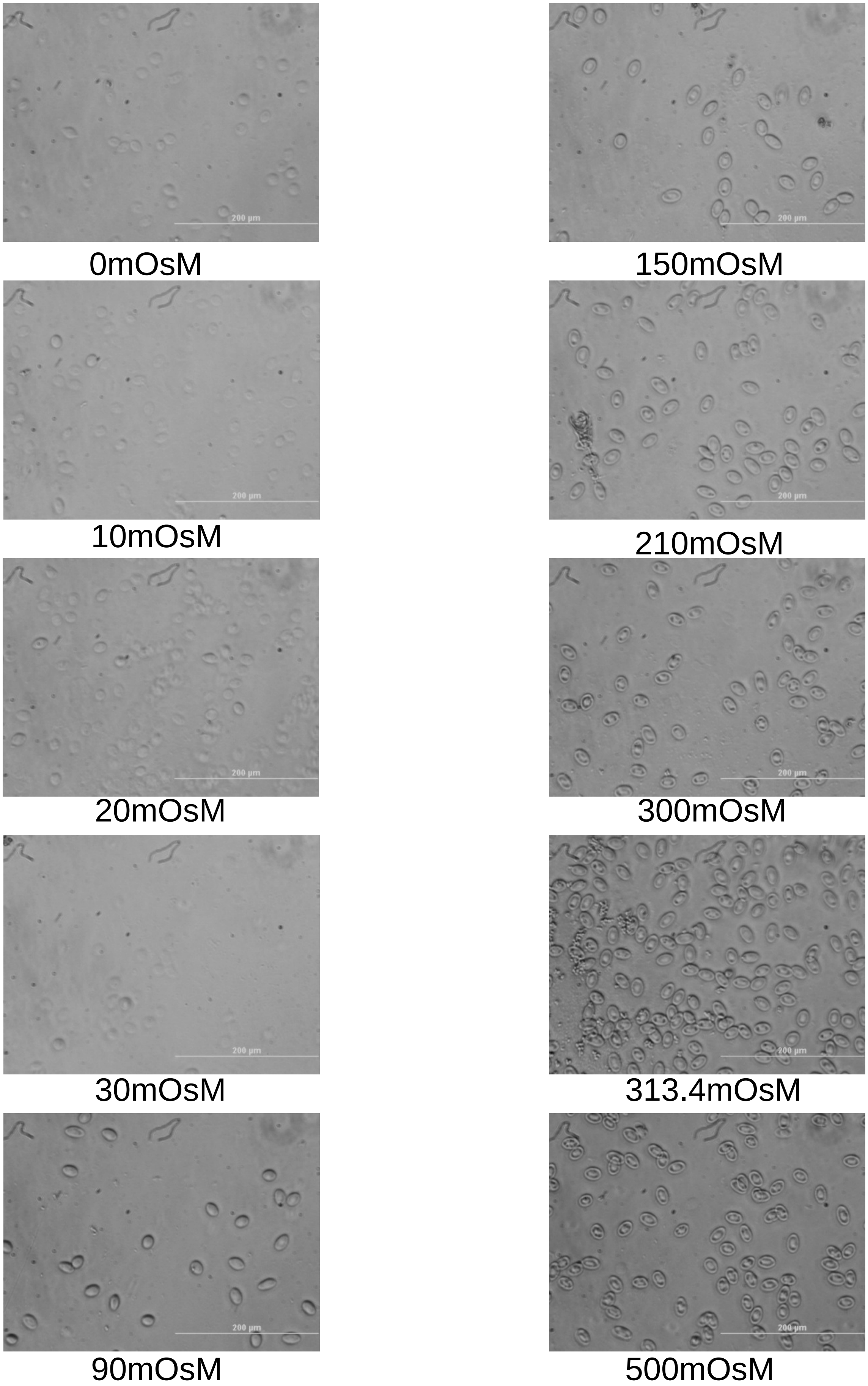

Figure S18: Chicken RBCs(With Trypsin Treatment)-Sucrose

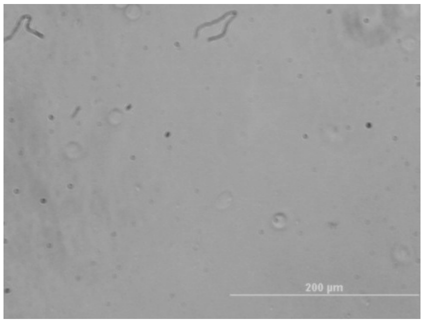

0mOsM

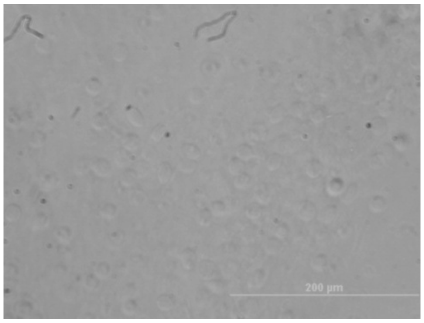

10mOsM

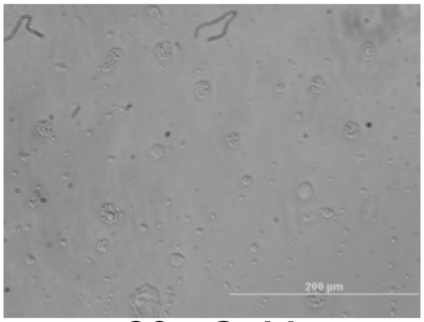

20mOsM

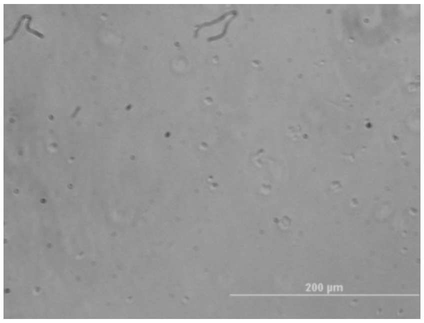

30mOsM

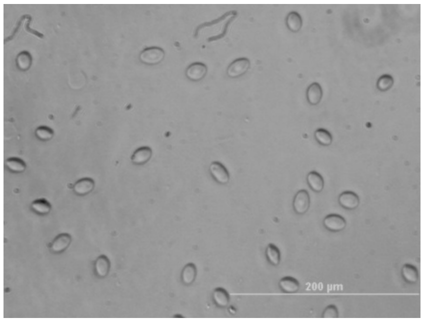

90mOsM

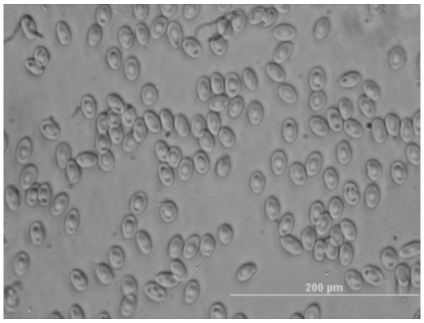

150mOsM

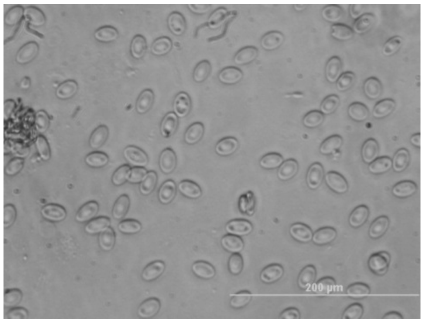

210mOsM

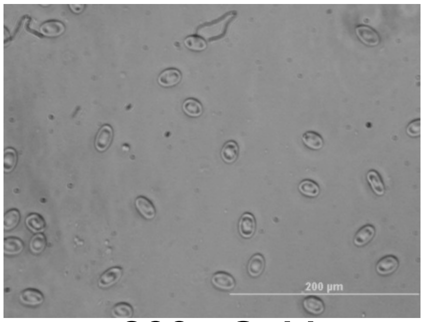

300mOsM

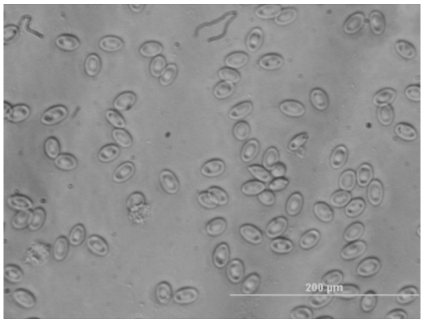

313.4mOsM

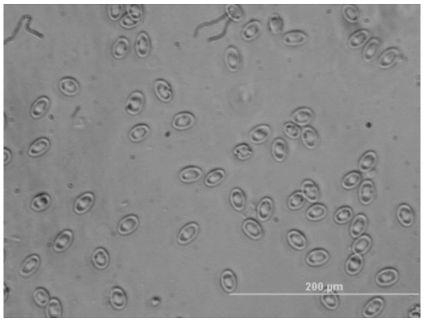

500mOsM

Figure S19

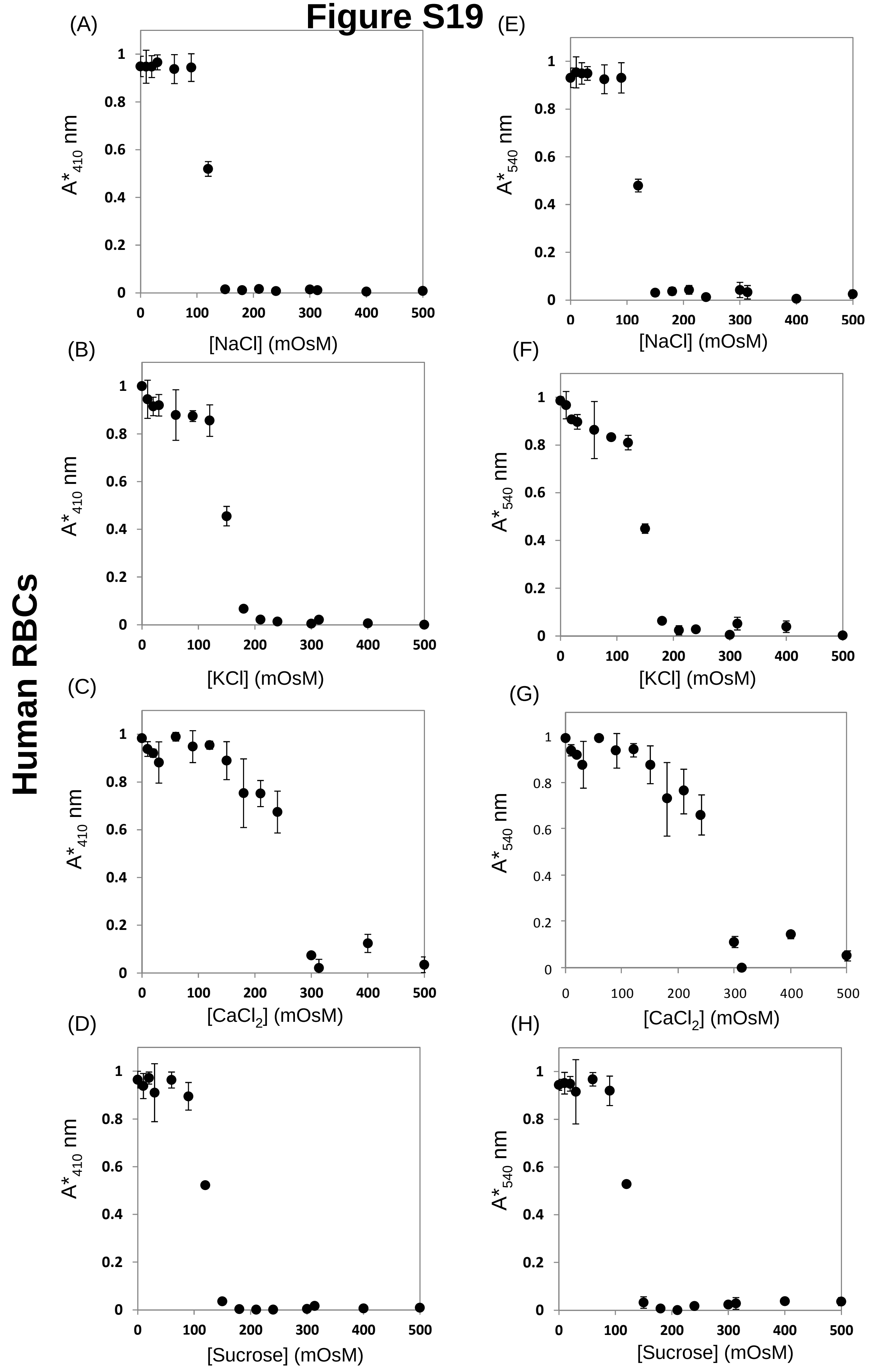
Human RBCs
(A)
(B)
(C)
(D)
(E)
(F)
(G)
(H)
A*$_{410}$ nm
A*$_{540}$ nm
[NaCl] (mOsM)
[KCl] (mOsM)
[CaCl$_2$] (mOsM)
[Sucrose] (mOsM)
0
0.2
0.4
0.6
0.8
1
0
100
200
300
400
500

Figure S20

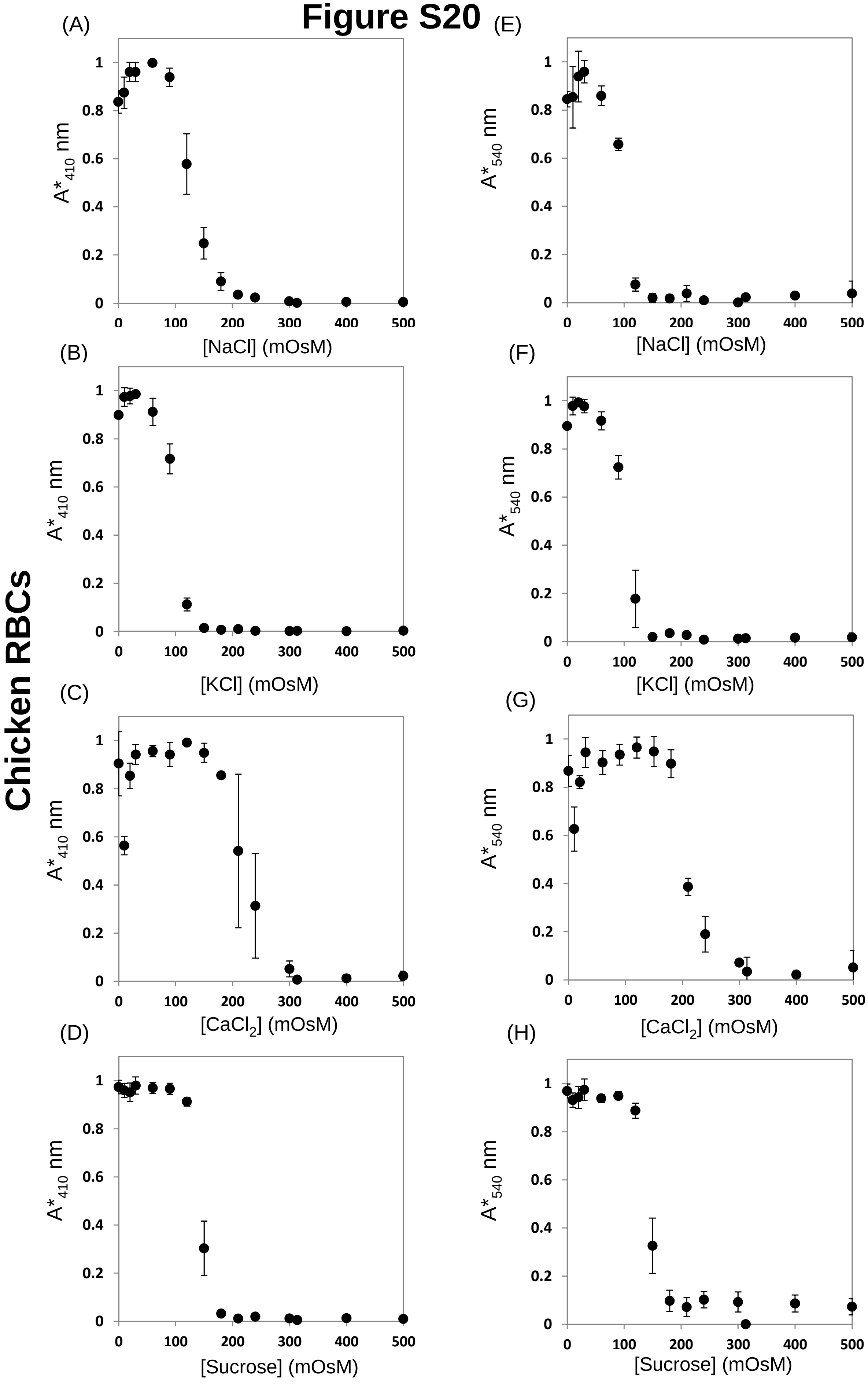

(A)
(B)
(C)
(D)
(E)
(F)
(G)
(H)
Chicken RBCs
A*410 nm
A*540 nm
[NaCl] (mOsM)
[KCl] (mOsM)
[CaCl2] (mOsM)
[Sucrose] (mOsM)
0
0.2
0.4
0.6
0.8
1
0
100
200
300
400
500

Figure S21

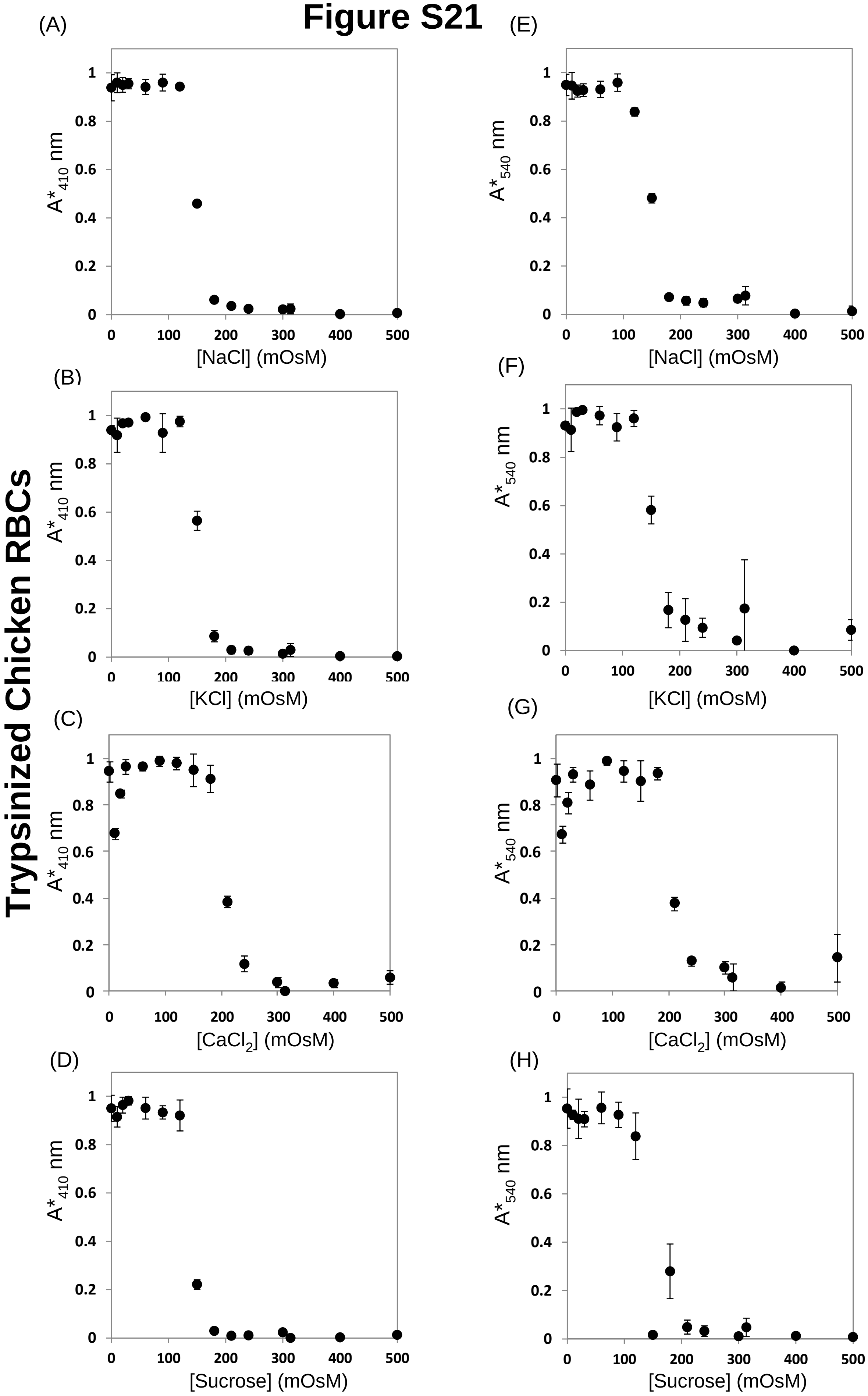

Trypsinized Chicken RBCs
(A)
(B)
(C)
(D)
(E)
(F)
(G)
(H)
A*410 nm
A*540 nm
[NaCl] (mOsM)
[KCl] (mOsM)
[CaCl2] (mOsM)
[Sucrose] (mOsM)
